# Supplementary material for: Molecular insights into the distinct signaling duration for the peptide-induced PTH1R activation
Source: Nat Commun. 2022 Oct 21;13:6276. doi: 10.1038/s41467-022-34009-x (PMC9586930; doi:10.1038/s41467-022-34009-x)
Supplement: Supplementary file 1 — Supplementary Information [file 41467_2022_34009_MOESM1_ESM.pdf]

Supplementary Information

Molecular insights into the distinct signaling duration for the peptide-induced PTH1R activation

Zhai et al.

Supplementary Figures 1-14

Supplementary Table 1-7

Supplementary Fig.1

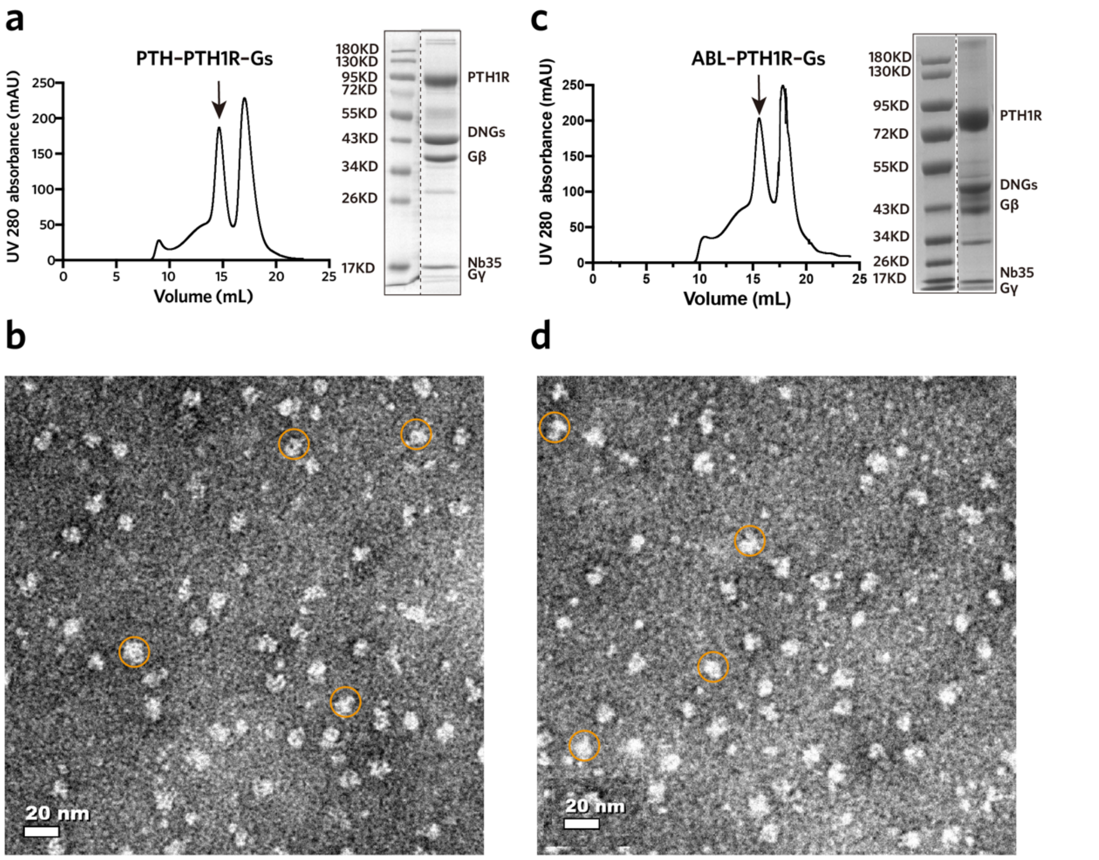

Supplementary Fig.1 Purification of PTH1R–Gs complexes.

a-b, Size exclusion chromatography (SEC) profile, SDS–PAGE and negative-staining EM analysis of the purified PTH–bound PTH1R–Gs complex. **c-d,** SEC profile, SDS–PAGE and negative-staining EM analysis of the purified ABL–bound PTH1R–Gs complex. Examples of the complex are circled in orange. The scale bar is 20 nm. Samples are prepared and repeated over three times. Source data are provided as a Source Data file.

Supplementary Fig.2

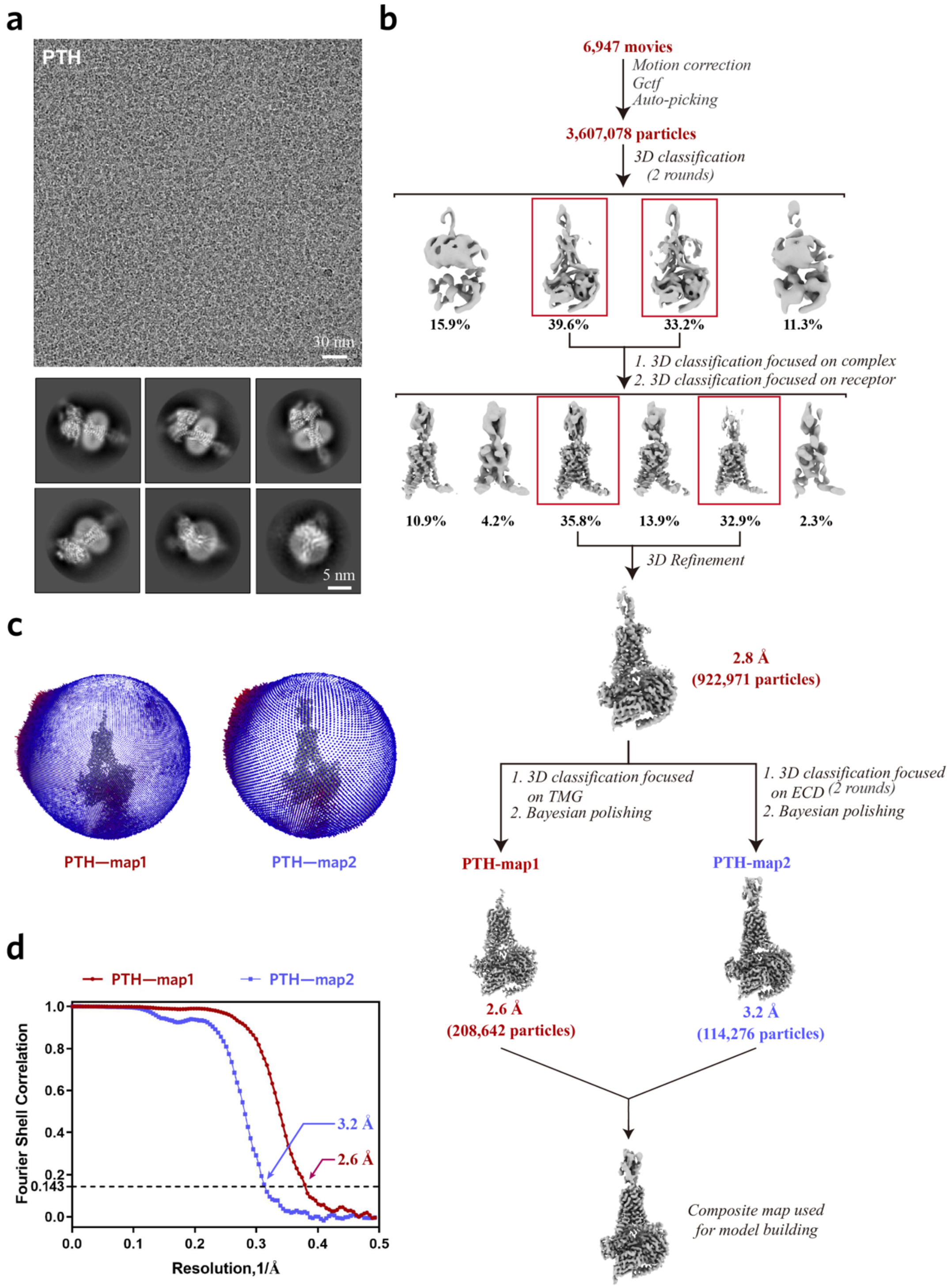

Supplementary Fig.2 Cryo-EM data processing of PTH–bound PTH1R–Gs complex.

a, Cryo-EM micrograph (scale bar: 30 nm) and 2D class averages (scale bar: 5 nm) of PTH–bound PTH1R–Gs complex. **b,** Flow chart of cryo-EM data processing (Details are given in the Method). **c,** Angular distribution of particles used in the final 3D

reconstruction. **d,** Fourier shell correlation (FSC) curves of the final refined map1 and map2.

Supplementary Fig.3

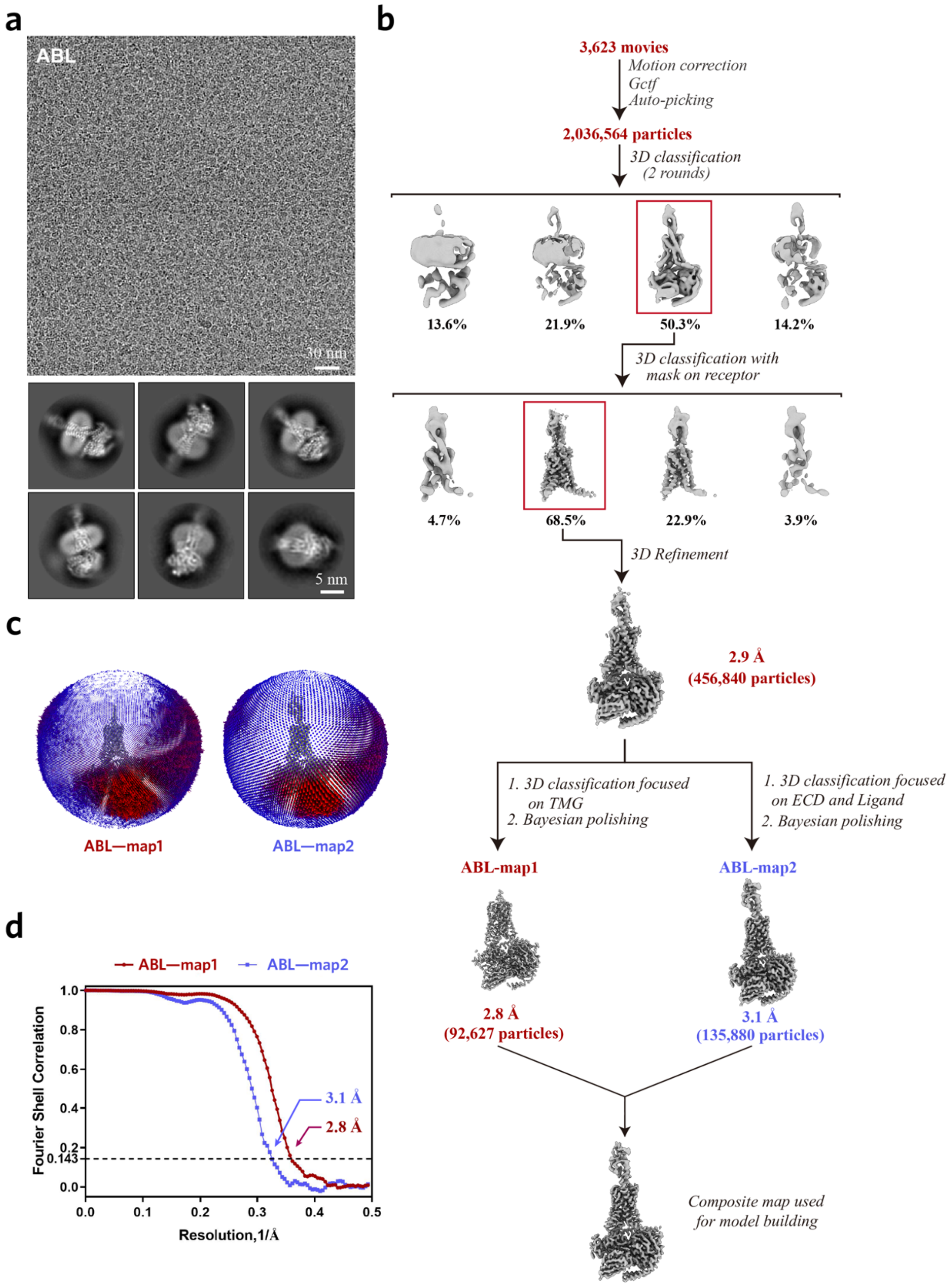

Supplementary Fig.3 Cryo-EM data processing of ABL–bound PTH1R–Gs complex.

a, Cryo-EM micrograph (scale bar: 30 nm) and 2D class averages (scale bar: 5 nm) of ABL–bound PTH1R–Gs complex. **b,** Flow chart of cryo-EM data processing (Details are given in the Method). **c,** Angular distribution of particles used in the final 3D

reconstruction. **d,** Fourier shell correlation (FSC) curves of the final refined map1 and map2.

Supplementary Fig.4

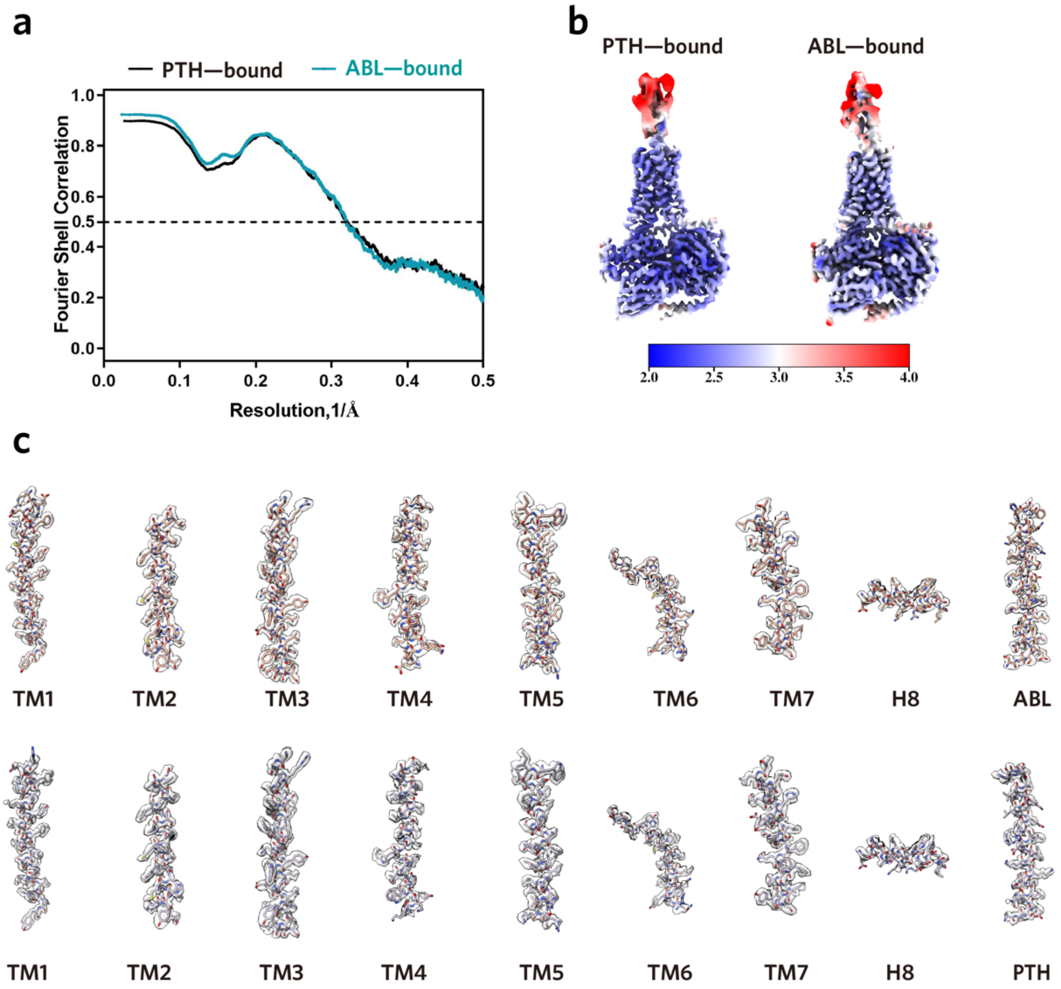

Supplementary Fig.4 Overall resolution and cryo-EM density analysis of the PTH1R–Gs complexes.

a, Fourier shell correlation curves of the model-vs-map. **b**, Cryo-EM maps coloured by local resolution (Å). **c**, Cryo-EM density maps and models are shown for all seven-transmembrane helices, helix 8 and peptide agonists of the PTH1R–Gs complexes.

Supplementary Fig.5

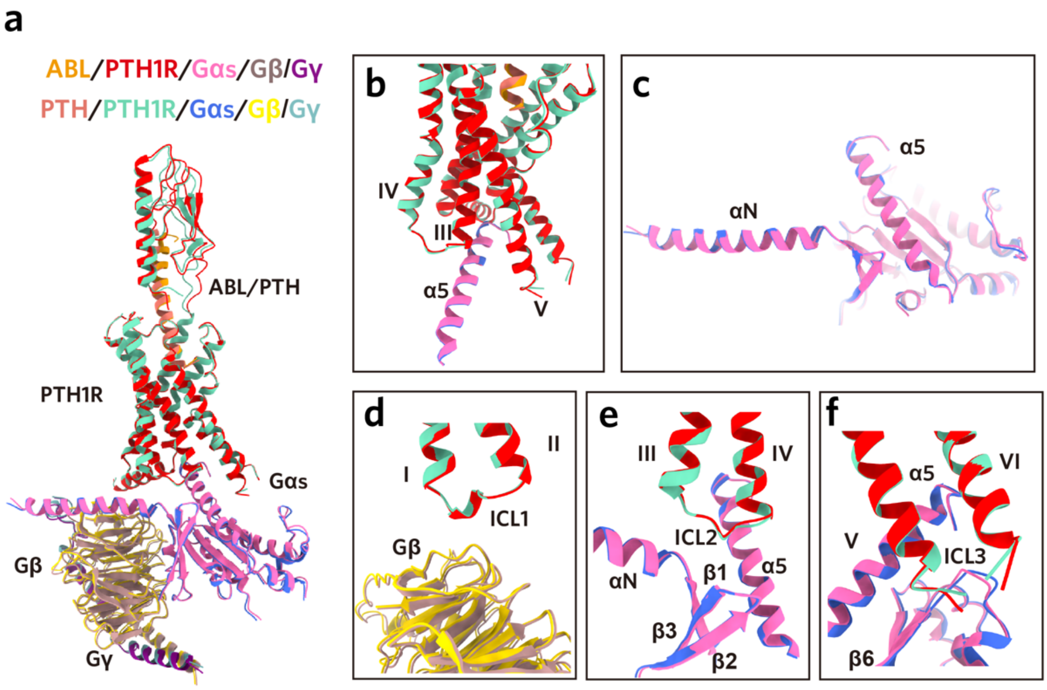

Supplementary Fig.5 Structural comparisons of the ABL and PTH–bound PTH1R-Gs complexes.

a, The ABL– and PTH–bound PTH1R-Gs complexes adopt highly similar global conformations. Structures were aligned by the TMD of PTH1R**. b-f,** Conserved interactions between PTH1R and Gs in the ABL– and PTH–bound complexes.

Supplementary Fig.6

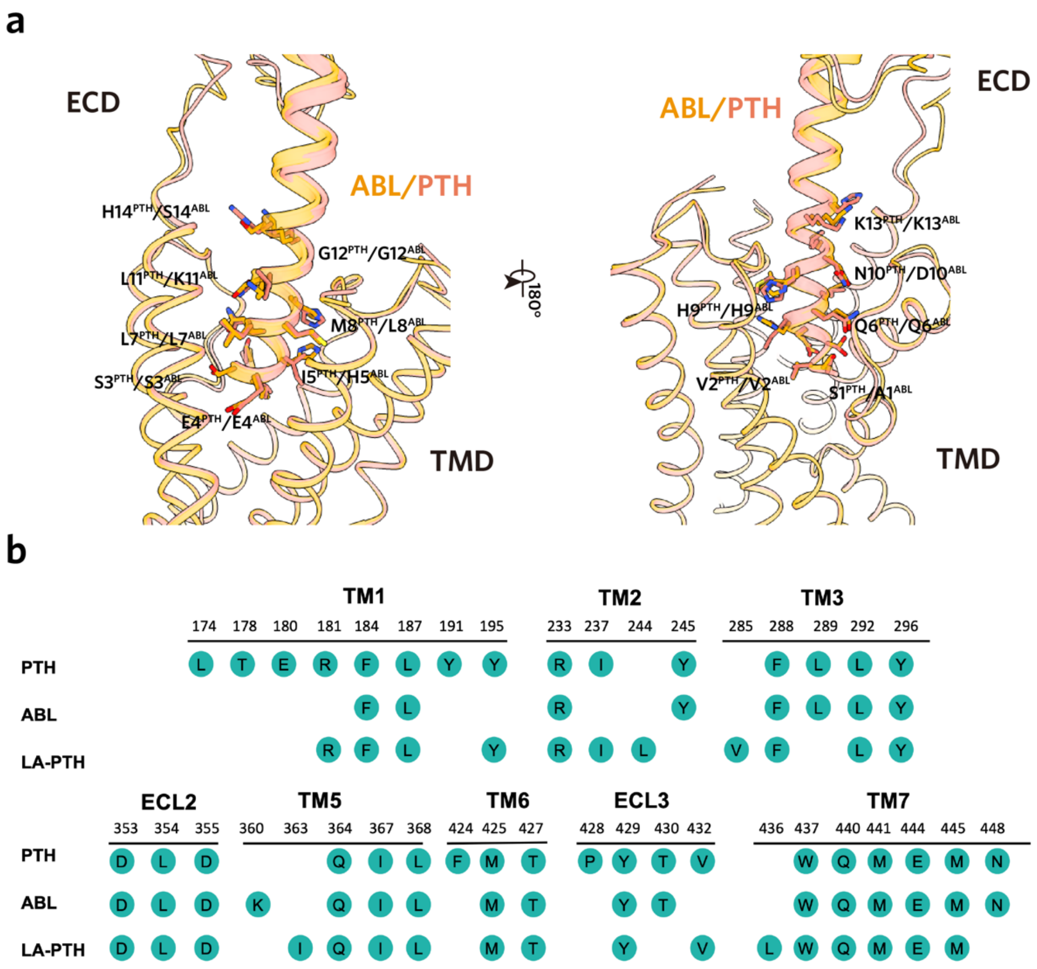

Supplementary Fig.6 Molecular recognition of peptide agonists by PTH1R.

a, The N-terminal portions of PTH and ABL show similar binding mode within the TM core of PTH1R. **b**, Detail interactions of PTH1R with the N-terminal portions of three peptide agonists (PTH, ABL and LA-PTH). Interactions between LA-PTH and PTH1R were analyzed by our previously reported structure (PDB 6NBF). Mutagenesis studies for the residues of the receptor that make differential contacts with the three peptides using cAMP signaling assays.

Supplementary Fig.7

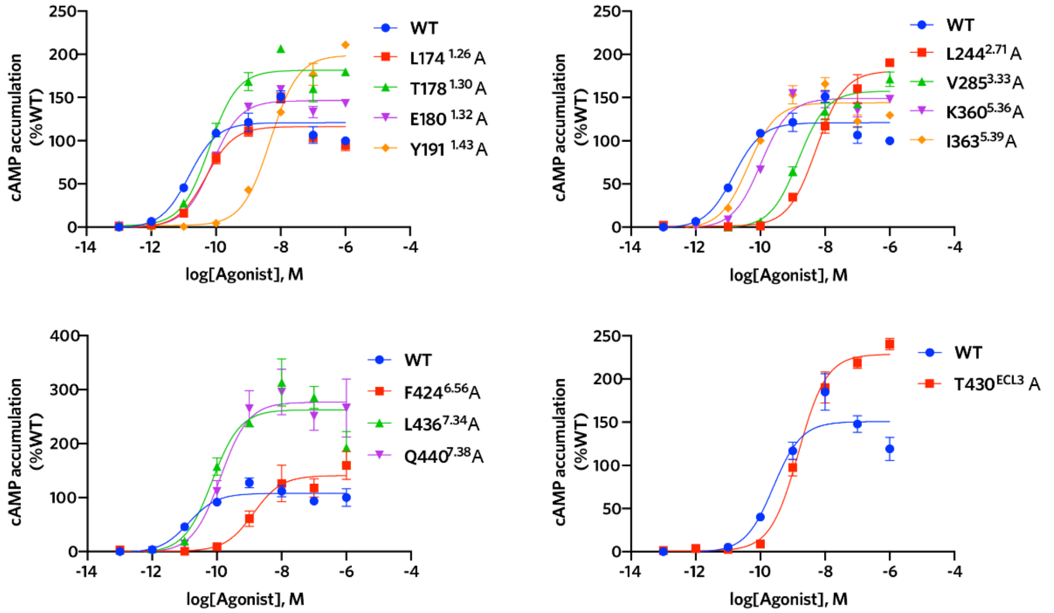

Supplementary Fig.7 ABL–induced cAMP accumulation of wild-type and mutant PTH1R.

Dose-response curves of cAMP accumulation measured by Glosensor assay. The data were analyzed using the 'log(agonist) vs. response-Variable slope (four parameters)' function in Graphpad Prism. All data are presented as mean values ± standard error of measurement (SEM) from three independent experiments performed in triplicate. Source data are provided as a Source Data file.

Supplementary Fig.8

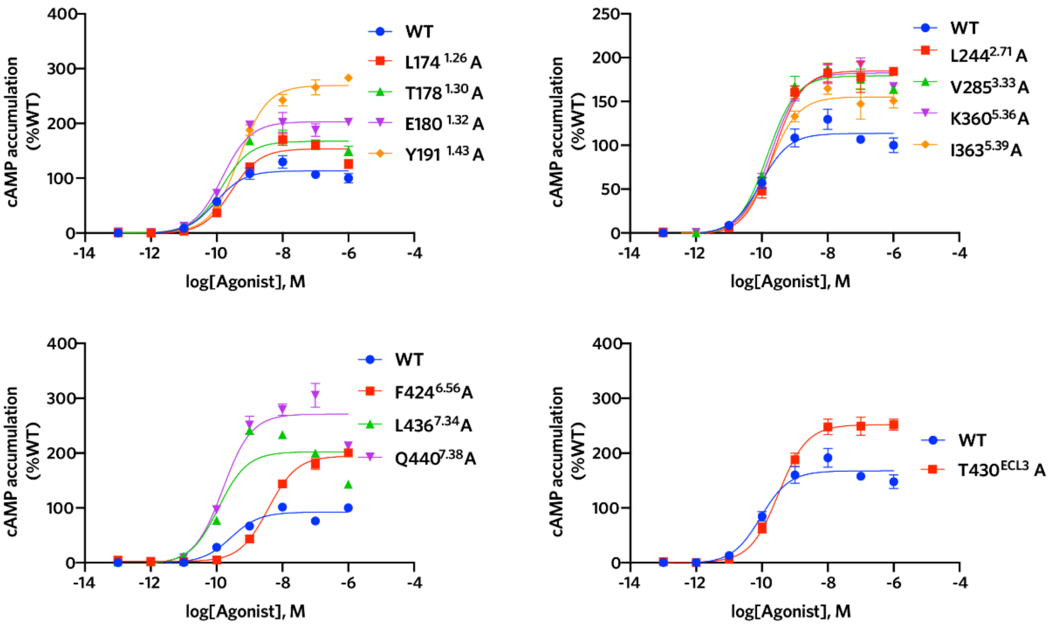

Supplementary Fig.8 PTH–induced cAMP accumulation of wild-type and mutant PTH1R.

Dose-response curves of cAMP accumulation measured by Glosensor assay. The data were analyzed using the 'log(agonist) vs. response-Variable slope (four parameters)' function in Graphpad Prism. All data are presented as mean values ± standard error of measurement (SEM) from three independent experiments performed in triplicate. Source data are provided as a Source Data file.

Supplementary Fig.9

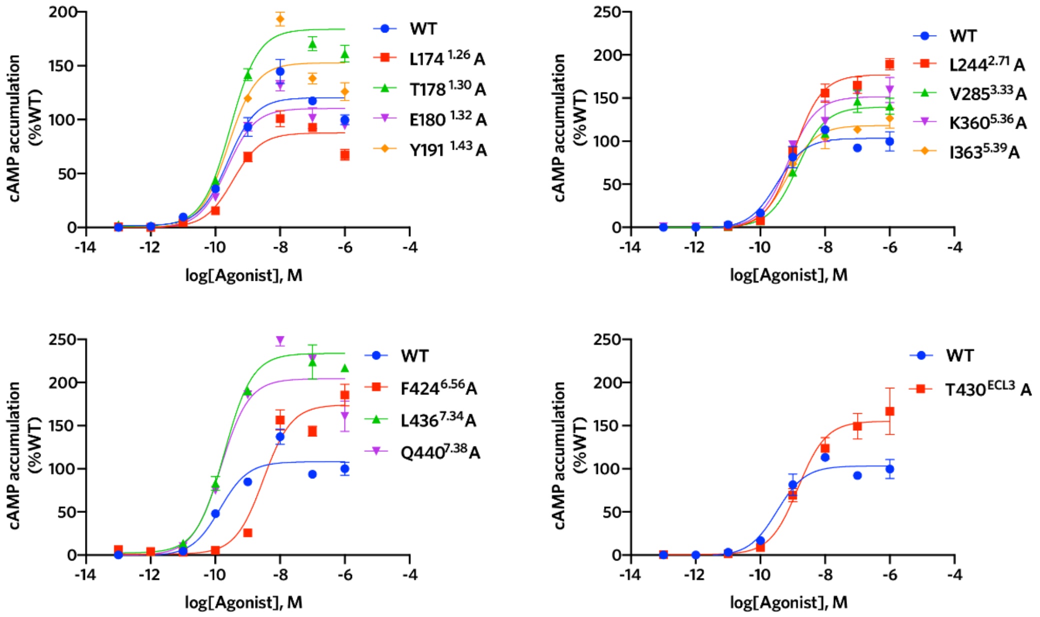

Supplementary Fig.9 LA-PTH–induced cAMP accumulation of wild-type and mutant PTH1R.

Dose-response curves of cAMP accumulation measured by Glosensor assay. The data were analyzed using the ‘log(agonist) vs. response-Variable slope (four parameters)’ function in Graphpad Prism. All data are presented as mean values ± standard error of measurement (SEM) from three independent experiments performed in triplicate. Source data are provided as a Source Data file.

Supplementary Fig.10

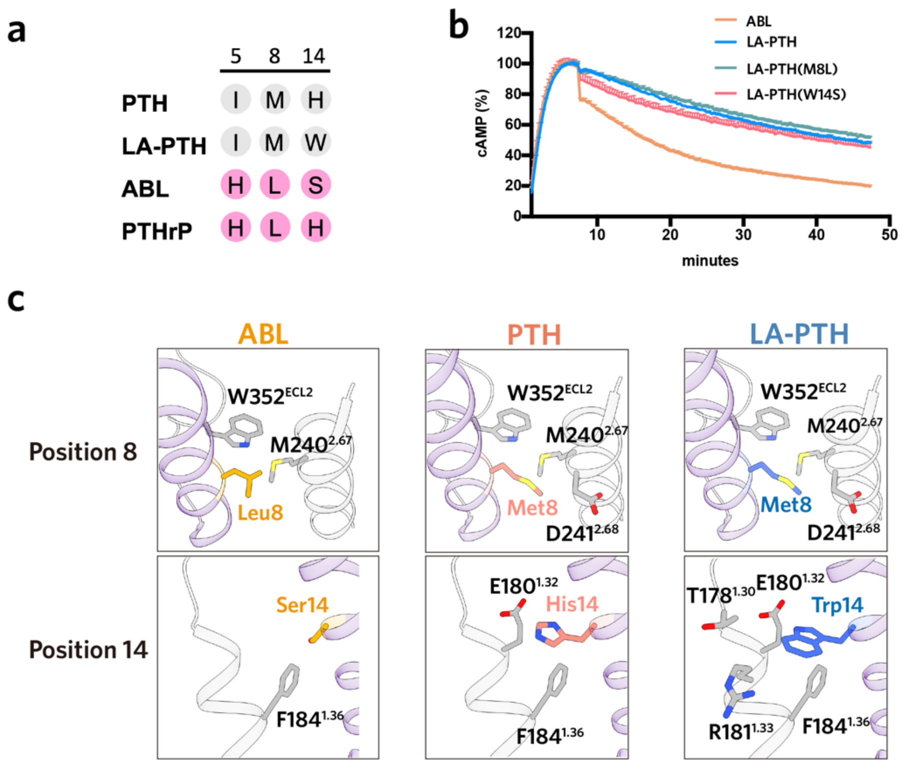

Supplementary Fig.10 Role of the N-terminal residues of LA-PTH in the prolonged cAMP signaling.

a, Sequence alignment of the residues 5, 8 and 14 in the N-terminal portion of peptide agonists. **b**, Effects of mutations in residues 8 and 14 of LA-PTH on its induced prolonged cAMP signaling. **c**, The potential interactions of residues in position 8 and 14 of peptide agonists with the R_0 state receptor (PDB 6FJ3). The potential interactions were analyzed based on the template of ePTH-bound PTH1R. Source data are provided as a Source Data file.

Supplementary Fig.11

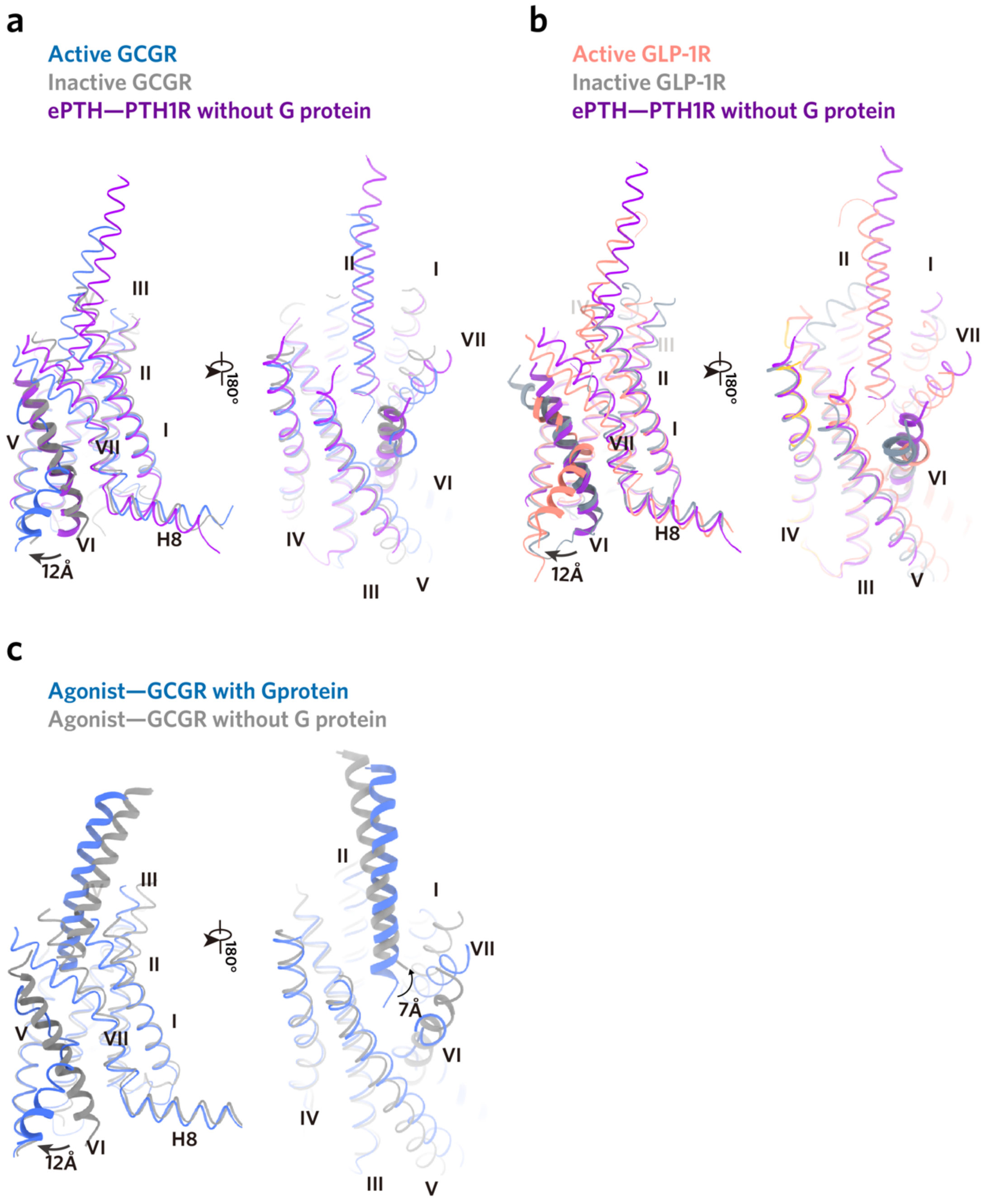

Supplementary Fig.11 The ePTH–bound PTH1R is probably a representative "intermediate state" in class B1 GPCRs.

a, Structural comparison of the ePTH–PTH1R (PDB 6FJ3) with active (PDB 6LMK) and inactive GCGR (PDB 5XEZ). **b**, Structural comparison of the ePTH–PTH1R (PDB 6FJ3) with active (PDB 6VCB) and inactive GLP-1R (PDB 6LN2). **c**, Structural comparison of the peptide–bound GCGR with (PDB 6LMK) and without G-protein (PDB 5YQZ).

Supplementary Fig.12

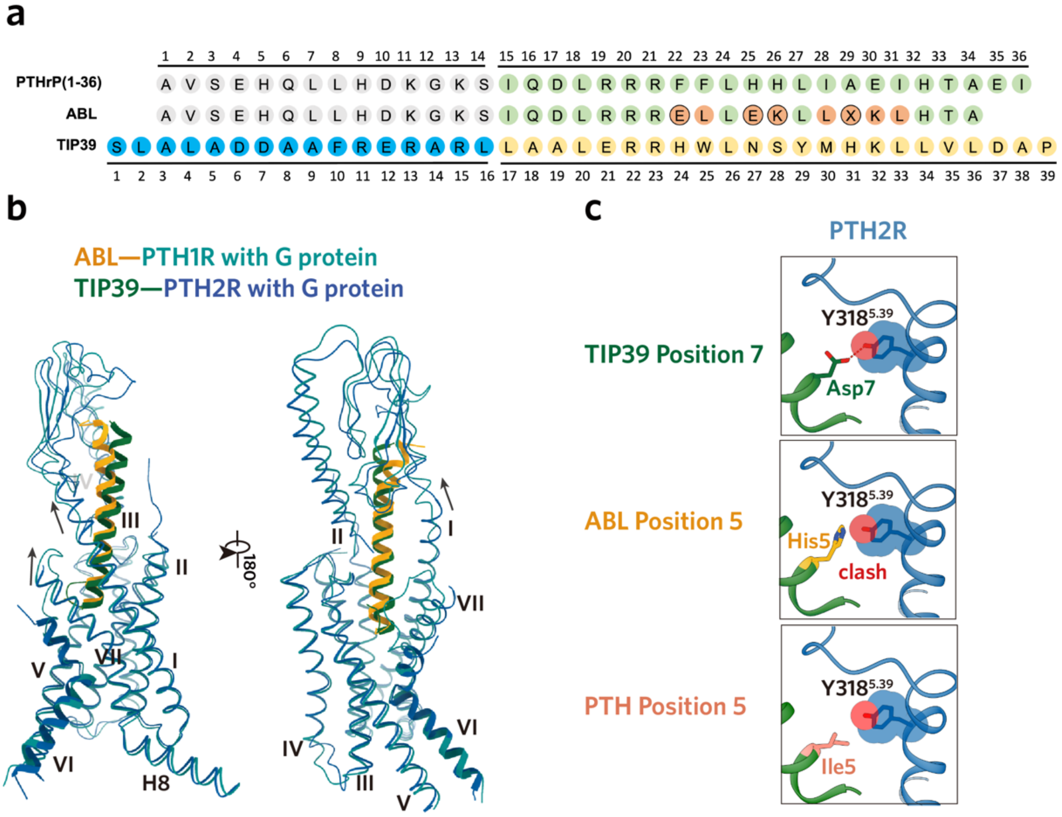

Supplementary Fig.12 Role of the divergent residue 5 between PTH and PTHrP in the recognition of PTH1R and PTH2R.

a, Sequence alignment of the PTHrP, ABL and TIP39. **b**, Structural comparisons of the ABL–bound PTH1R with TIP39–bound PTH2R (PDB 7F16). **c**, The divergent residue 5 between PTH and PTHrP makes differential interactions with PTH2R, which may explain the weak actions between PTHrP and PTH2R.

Supplementary Fig.13

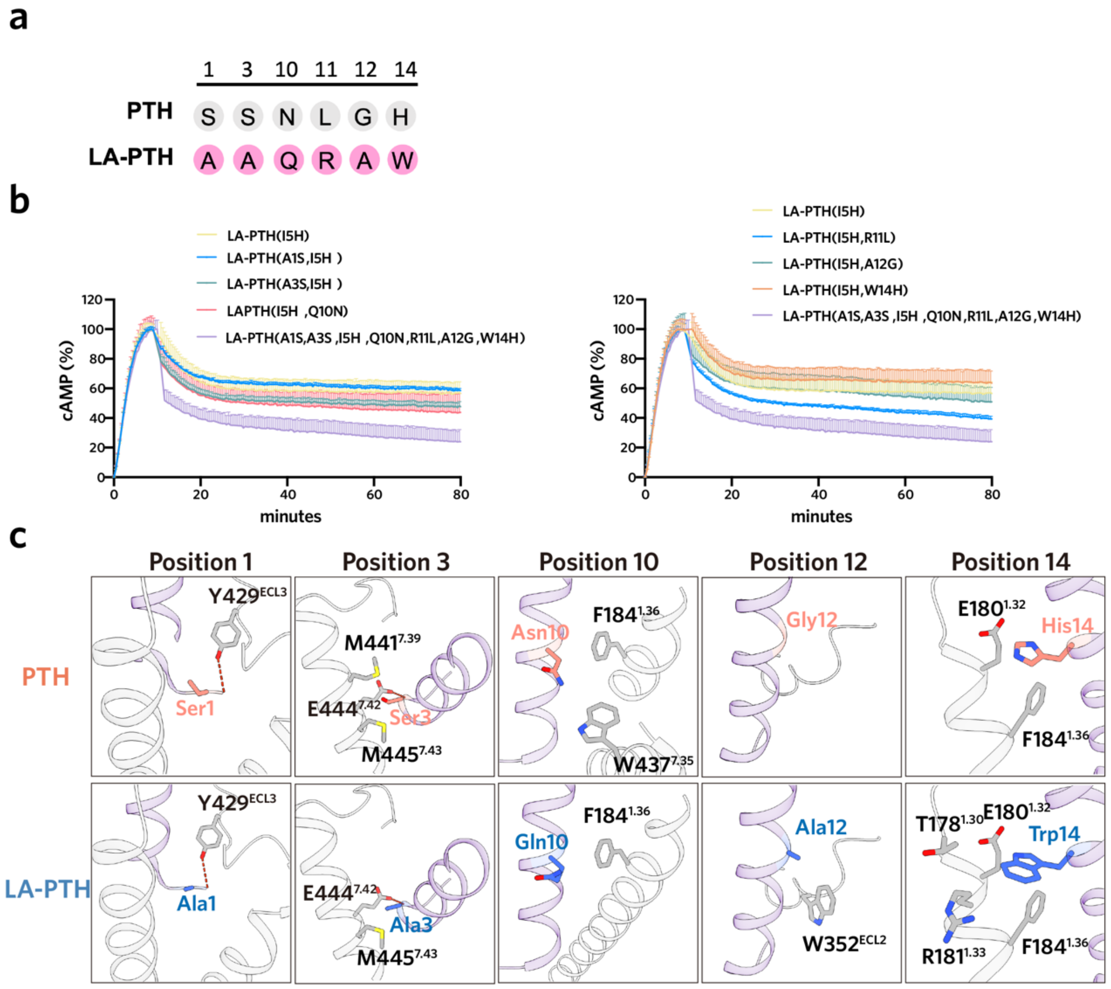

Supplementary Fig.13 Role of "M" substitutions in LA-PTH–induced prolonged cAMP signaling.

a, Sequence alignment of the divergences in the N-terminal portion of LA-PTH and PTH. "M" substitutions in LA-PTH are colored pink. **b**, Effects of mutations in the "M" substitutions of LA-PTH on its induced prolonged cAMP signaling. **c**, The potential interactions of the "M" substitutions in LA-PTH with the R_0 state receptor (PDB 6FJ3). The potential interactions were analyzed based on the template of ePTH-bound PTH1R. Source data are provided as a Source Data file.

Supplementary Fig.14

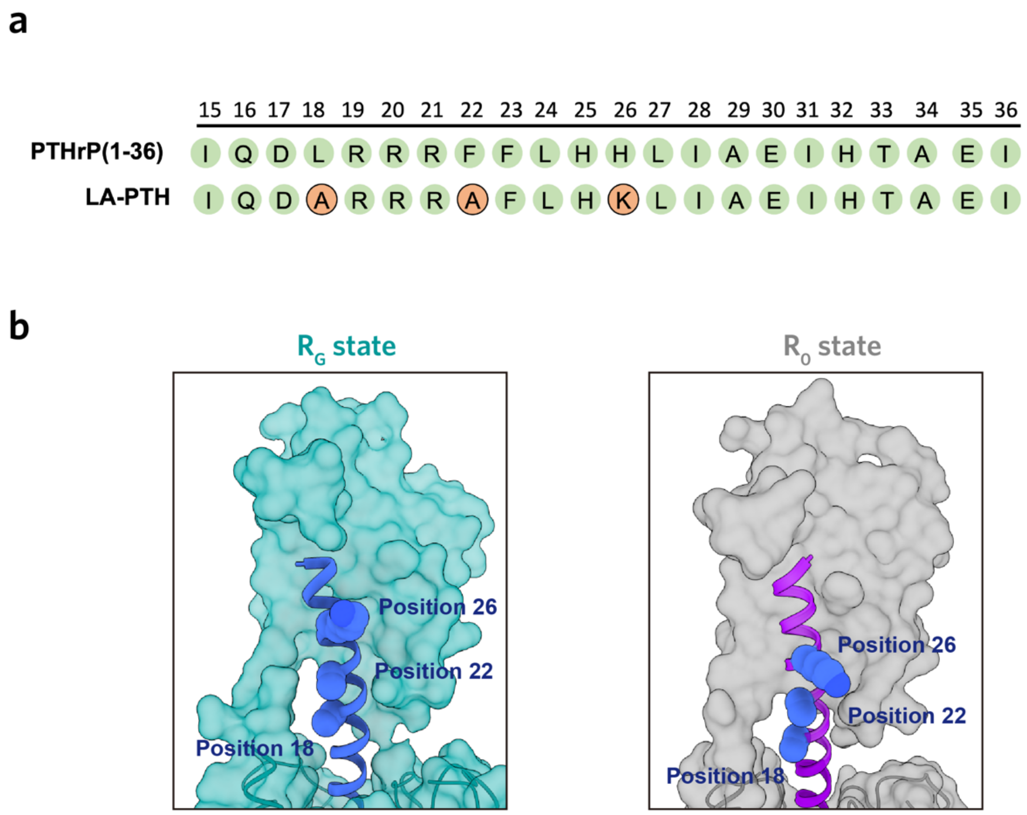

Supplementary Fig.14 Sequence and structural analysis of the three residue substitutions in the C-terminal portion of LA-PTH.

a, Sequence alignment between the C-terminal portion of LA-PTH and PTHrP. The residue discrepancies in the C-terminal portion of the two peptides are colored orange.

b, The three residues (position 18, 22 and 26) in the C-terminal portion of peptide are located at the solvent-accessible side of the peptide agonists for both R_G (PDB 6NBF) and R_0 states (PDB 6FJ3) of PTH1R.

Supplementary Table.1 Cryo-EM data collection, refinement and validation statistics

	PTH–PTH1R–DNGαs (EMDB-33590) (PDB-7Y36)	ABL–PTH1R–DNGαs (EMDB-33588) (PDB-7Y35)
**Data collection and processing**		
Magnification	4,9310	4,9310
Voltage (kV)	300	300
Electron exposure (e^{-}/Å^2)	64	64
Defocus range (μm)	-0.5 ~ -2.5	-0.5 ~ -2.5
Pixel size (Å)	1.014	1.014
Symmetry imposed	C1	C1
Initial particle projections	3,607,078	2,036,564
Final particle projections	on TMG:208,642<br>on ECD:114,276	on TMG: 92,627<br>on ECD:135,880
Map resolution (Å)	2.8	2.9
FSC threshold	0.143	0.143
Map resolution range (Å)	2.5-5.0	2.5-5.0
**Refinement**		
Initial model used	6NBF	6NBF
Model resolution (Å)	3.2	3.1
FSC threshold	0.5	0.5
Map sharpening method	DeepEMhancer	DeepEMhancer
Model composition		
Non-hydrogen atoms	9218	9203
Protein residues	1153	1152
Ligand	1	1
B factors (Å^2)		
Protein	99.58	94.67
Ligand	110.91	95.45
R.m.s. deviations		
Bond lengths (Å)	0.007	0.010
Bond angles (°)	0.732	0.800
Validation		
MolProbity score	1.68	1.54
Clashscore	7.11	5.09
Rotamer outliers (%)	0.00	0.00
Ramachandran plot		
Favored (%)	95.84	96.01
Allowed (%)	4.16	3.99
Disallowed (%)	0	0

Supplementary Table.2 Interactions of PTH with PTH1R.

Residues within 4 Å are shown.

PTH	PTH1R	Interaction
Ser1	Phe424$^{6.56}$	Hydrogen bond
	Met425$^{6.57}$	
	Thr427$^{6.59}$	
	Pro428^{ECL3}	Polar interaction
	Tyr429^{ECL3}	Van der waals force
Val2	Tyr296$^{3.44}$	Van der waals force
	Gln364$^{5.40}$	Polar interaction
	Ile367$^{5.43}$	Hydrophobic interaction
Ser3	Gln440$^{7.38}$	Polar interaction
	Glu444$^{7.42}$	
	Met441$^{7.39}$	Van der waals force
	Met445$^{7.43}$	
Glu4	Arg233$^{2.60}$	Electrostatic interaction
	Ile237$^{2.64}$	Van der waals force
	Phe288$^{3.36}$	
	Tyr195$^{1.47}$	Hydrogen bond
	Asn448$^{7.46}$	Hydrogen bond
Ile5	Leu289$^{3.37}$	Hydrophobic interaction
	Leu292$^{3.40}$	
	Gln364$^{5.40}$	Van der waals force
Gln6	Thr430^{ECL3}	Polar interaction
	Trp437$^{7.35}$	Van der waals force
	Gln440$^{7.38}$	Hydrogen bond
	Met441$^{7.38}$	Hydrophobic interaction
	Tyr429^{ECL3}	
Leu7	Leu187$^{1.39}$	Hydrophobic interaction
	Met441$^{7.38}$	Van der waals force
	Met445$^{7.43}$	
Met8	Tyr245$^{2.72}$	Van der waals force
	Asp353^{ECL2}	
His9	Asp353^{ECL2}	Electrostatic interaction
	Tyr429^{ECL3}	Van der waals force
	Ser354^{ECL2}	
	Ser355^{ECL2}	
Asn10	Val432^{ECL2}	Van der waals force
	Trp437$^{7.35}$	
	Phe184$^{1.63}$	

Leu11	Phe184$^{1.63}$	Hydrophobic interaction
	Tyr245$^{2.72}$	
Gly12	Leu354^{ECL3}	Hydrophobic interaction
Lys13	Leu354^{ECL3}	Van der waals force
His14	Glu180$^{1.32}$	Electrostatic interaction
	Arg181$^{1.33}$	Van der waals force
	Phe184$^{1.36}$	

Supplementary Table.3 Interactions of ABL with PTH1R.

Residues within 4 Å are shown.

ABL	PTH1R	Interaction
Ala1	Leu368$^{5.44}$	Hydrophobic interaction
	Met425$^{6.57}$	Hydrogen bond
	Thr427$^{6.59}$	Polar interaction
	Tyr429^{ECL3}	Van der waals force
Val2	Tyr296$^{3.44}$	Van der waals force
	Gln364$^{5.40}$	Polar interaction
	Ile367$^{5.43}$	Hydrophobic interaction
Ser3	Met441$^{7.39}$	Polar interaction
	Glu444$^{7.42}$	
	Met445$^{7.43}$	Van der waals force
Glu4	Arg233$^{2.60}$	Salt bridge
	Tyr296$^{3.44}$	Polar interaction
	Met445$^{7.43}$	Hydrophobic interaction
	Asn448$^{7.46}$	Hydrogen bond
His5	Leu289$^{3.37}$	Van der waals force
	Lys360$^{5.36}$	
	Gln364$^{5.40}$	Hydrogen bond
	Tyr429^{ECL3}	Polar interaction
Gln6	Tyr429^{ECL3}	Van der waals force
	Thr430 ^{ECL3}	Polar interaction
	Trp437$^{7.35}$	Van der waals force
	Gln440$^{7.38}$	Polar interaction
	Met441$^{7.39}$	Hydrophobic interaction
Leu7	Phe184$^{1.36}$	Hydrophobic interaction
	Leu187$^{1.39}$	
	Trp437$^{7.35}$	Van der waals force
	Met445$^{7.43}$	Hydrophobic interaction
Leu8	Tyr245$^{2.72}$	Polar interaction
His9	Asp353^{ECL2}	Polar interaction
	Ser355 ^{ECL2}	
	Tyr429^{ECL3}	

Asp10	$Phe184^{1.63}$	Van der waals force
	$Typ437^{7.35}$	Polar interaction
	$Met441^{7.39}$	Van der waals force
Lys11	$Phe184^{1.36}$	Van der waals force
	$Tyr245^{2.72}$	
Lys13	$Leu354^{ECL2}$	Hydrophobic interaction

Supplementary Table.4 PTH–induced cAMP accumulation of wild-type and mutant PTH1R.

	ΔpEC_{50}±SEM[a,b]	Span±SEM[a,b]	Sample size	Expression (%WT)
WT	0	125.53±6.27	3	100
L174$^{1.26}$A	-0.53±0.09****	116.27±19.65	3	135.1±12.67
T178$^{1.30}$A	-0.21±0.05^{ns}	147.23±17.05	3	70.43±3.37
E180$^{1.32}$A	-0.11±0.07^{ns}	193.57±39.96	3	99.65±4.01
Y191$^{1.43}$A	-0.84±0.08****	164.77±14.05	3	124.6±9.82
L244$^{2.71}$A	-0.48±0.05****	150.17±18.04	3	99.96±6.93
V285$^{3.33}$A	-0.15±0.04^{ns}	152.43±15.43	3	102.6±7.87
K360$^{5.36}$A	-0.14±0.10^{ns}	135.06±20.89	3	133.7±9.52
I363$^{5.39}$A	-0.28±0.04*	180.23±7.67	3	69.63±2.06
F424$^{6.56}$A	-1.43±0.05****	143.97±16.16	3	111.1±24.91
T430^{ECL3}A	-0.32±0.03**	174.77±10.12	3	98.22±20.13
L436$^{7.34}$A	0.06±0.04^{ns}	220.70±25.60	3	104.2±28.38
Q440$^{7.38}$A	-0.16±0.03^{ns}	297.80±37.90	3	145.5±30.32

[a]Data shown are means ± SEM from at least three independent experiments performed in technical triplicate. $^{ns}P > 0.01$, $^{*}P < 0.05$, $^{**}P < 0.01$, $^{***}P < 0.001$ and $^{****}P < 0.0001$ by one-way ANOVA followed by Dunnett's post-test, compared with the response of theWT. [b]The span is defined as the window between the maximal response (Emax) and the vehicle (no PTH). Source data are provided as a Source Data file.

Supplementary Table.5 ABL–induced cAMP accumulation of wild-type and mutant PTH1R.

	ΔpEC_{50}±SEM[a,b]	Span±SEM[a,b]	Sample size	Expression (% WT)
WT	0	121.80±3.30	3	100
L174$^{1.26}$A	-0.52±0.09****	113.79±10.36	3	135.1±12.67
T178$^{1.30}$A	-0.73±0.06****	132.49±24.86	3	70.43±3.37
E180$^{1.32}$A	-0.55±0.03****	134.23±13.07	3	99.65±4.01
Y191$^{1.43}$A	-2.49±0.07****	230.23±16.63	3	124.6±9.82
L244$^{2.71}$A	-2.54±0.04****	224.50±22.26	3	99.96±6.93
V285$^{3.33}$A	-2.06±0.1****	173.37±7.80	3	102.6±7.87
K360$^{5.36}$A	-1.13±0.05****	130.30±10.66	3	133.7±9.52
I363$^{5.39}$A	-0.57±0.08****	151.93±9.20	3	69.63±2.06
F424$^{6.56}$A	-2.01±0.04****	153.20±9.11	3	111.1±24.91
T430^{ECL3}A	-0.8±0.03****	204.13±13.19	3	98.22±20.13
L436$^{7.34}$A	-0.63±0.09****	225.53±30.07	3	104.2±28.38
Q440$^{7.38}$A	-1.05±0.04****	289.77±25.27	3	145.5±30.32

[a]Data shown are means ± SEM from at least three independent experiments performed in technical triplicate. $^{ns}P > 0.01$, $^{*}P < 0.05$, $^{**}P < 0.01$, $^{***}P < 0.001$ and $^{****}P < 0.0001$ by one-way ANOVA followed by Dunnett's post-test, compared with the response of theWT. [b]The span is defined as the window between the maximal response (Emax) and the vehicle (no ABL). Source data are provided as a Source Data file.

Supplementary Table.6 LA-PTH–induced cAMP accumulation of wild-type and mutant PTH1R.

	ΔpEC_{50}±SEM[a,b]	Span±SEM[a,b]	Sample size	Expression (%WT)
WT	0	114.00±3.67	3	100
L174$^{1.26}$A	-0.20±0.03 ^{ns}	93.27±3.07	3	135.1±12.67
T178$^{1.30}$A	-0.05±0.02^{ns}	133.60±23.62	3	70.43±3.37
E180$^{1.32}$A	0.03±0.04^{ns}	158.60±26.43	3	99.65±4.01
Y191$^{1.43}$A	-0.09±0.04 ^{ns}	148.60±7.84	3	124.6±9.82
L244$^{2.71}$A	-0.39±0.02***	167.07±18.33	3	99.96±6.93
V285$^{3.33}$A	-0.42±0.05***	170.70±21.34	3	102.6±7.87
K360$^{5.36}$A	-0.08±0.09^{ns}	133.83±9.72	3	133.7±9.52
I363$^{5.39}$A	-0.05±0.19 ^{ns}	118.06±12.99	3	69.63±2.06
F424$^{6.56}$A	-1.16±0.1****	143.97±16.16	3	111.1±24.91
T430^{ECL3}A	-1.10±0.03****	174.33±11.17	3	98.22±20.13
L436$^{7.34}$A	0.01±0.07^{ns}	201.20±19.49	3	104.2±28.38
Q440$^{7.38}$A	-0.01±0.02^{ns}	289.33±25.51	3	145.5±30.32

[a]Data shown are means ± SEM from at least three independent experiments performed in technical triplicate. $^{ns}P > 0.01$, $^{*}P < 0.05$, $^{**}P < 0.01$, $^{***}P < 0.001$ and $^{****}P < 0.0001$ by one-way ANOVA followed by Dunnett's post-test, compared with the response of theWT. [b]The span is defined as the window between the maximal response (Emax) and the vehicle (no LA-PTH). Source data are provided as a Source Data file.

Supplementary Table 7. List of primer sequences used in this study

Oligonucleotide name	Oligonucleotide sequence (5'→3')	Cloning	Product
PTH1R-FL F	tcttctgcctggtattcgccGATGCAGATGACGTCATGACTAAAG	Homologous recombination	pfastbac-PTH1R-FL-LgBiT
PTH1R-FL R	tcgagtgtgaagacgaattcCATCACGGTTTCCCACTCTTCT		
Linear pfastbac F	GAATTCGTCTTCACACTCGAAGATT		
Linear pfastbac R	GGCGAATACCAGGCAGAAGATGTA		
PTH1R-FL F	ACAAGGACGATGATGACAAGGATGCAGATGACGTCATGACTAAAG	Homologous recombination	pcDNA 3.0-PTH1R-FL
PTH1R-FL-R	tttaaacgggccctctactaCATCACGGTTTCCCACTCTTCT		
Linear pcDNA F	TAGTAGAGGGCCCGTTTAAACCC		
Linear pcDNA R	CTTGTCATCATCGTCCTTGTAGTCG		
PTH1R-FL F	aagtccttttccagggccctGATGCAGATGACGTCATGACTAAAG	Homologous recombination	pcDNA 3.0-PTH1R-FL-LgBiT
PTH1R-FL-LgBiT R	aaatcttcgagtgtgaagacCATCACGGTTTCCCACTCTTCT		
Linear pcDNA F	GTCTTCACACTCGAAGATTTCGTTG		
Linear pcDNA R	AGGGCCCTGGAAAAGGACT		
L174A F	TgcaACCAATGAGACTCGTGAACGGGAGGTGT	Site-directed mutagenesis	pcDNA 3.0-PTH1R-FL(L174A)
L174A R	GAGTCTCATTGGTtgcAAATTTGACACACTCGCTGTAGTTG		
T178A F	CAATGAGgcaCGTGAACGGGAGGTGTTTGACC		pcDNA 3.0-PTH1R-FL(T178A)
T178A R	GTTCACGtgcCTCATTGGTGAGAAATTTGACACAC		
E180A F	AATGAGACTCGTgcaCGGGAGGTGTTTGACCGC		pcDNA 3.0-PTH1R-FL(E180A)
E180A R	CGtgcACGAGTCTCATTGGTGAGAAATTTGAC		
Y191A F	CATGATTgcaACCGTGGGCTACTCCGTGTCCC		pcDNA 3.0-PTH1R-FL(Y191A)
Y191A R	CCACGGTtgcAATCATGGCCAGGCGGTCAAAC		
L244A F	ACGCTGTGgcaTACTCTGGCGCCACGCTTGAT		pcDNA 3.0-PTH1R-FL(L244A)
L244A R	AGAGTAtgcCACAGCGTCCTTGACGAAGATGC		
V285A F	GTGGCTgcaACCTTCTTCCTTTACTTCCTGGC		pcDNA 3.0-PTH1R-FL(V285A)
V285A R	AAGAAGGTtgcAGCCACCCTGCAGCCCGCGTA		
K360A F	GGAACAAAgcaTGGATCATCCAGGTGCCCATC		pcDNA 3.0-PTH1R-FL(K360A)
K360A R	GATCCAtgcTTTGTTCCCGGAGCTCAAGTCCC		
I363A F	AAGTGGATCgcaCAGGTGCCCATCCTGGCCTC		pcDNA 3.0-PTH1R-FL(I363A)
I363A R	ACCTGtgcGATCCACTTTTTGTTCCCGGAGCT		
F424A F	ACATTGTCgcaGCCACACCATACACCGAGGTC		pcDNA 3.0-PTH1R-FL(F424A)
F424A R	TGTGGCtgcGACAATGTAGTGGACGCCAAAGA		
T430A F	ACCATACgcaGAGGTCTCAGGGACGCTCTGGC		pcDNA 3.0-PTH1R-FL(T430A)
T430A R	AGACCTCtgcGTATGGTGTGGCCATGAAGACA		
L436A F	gcaTGGCAAGTCCAGATGCACTATGAGATGCT		pcDNA 3.0-PTH1R-FL(L436A)
L436A R	ATCTGGACTTGCCAtgcCGTCCCTGAGACCTCGGTG		
Q440A F	GCAAGTCgcaATGCACTATGAGATGCTCTTCAACTC		pcDNA 3.0-PTH1R-FL(Q440A)
Q440A R	AGTGCATtgcGACTTGCCAGAGCGTCCCTGAG		
