# Supplementary material for: Molecular insights into the distinct signaling duration for the peptide-induced PTH1R activation
Source: Nat Commun. 2022 Oct 21;13:6276. doi: 10.1038/s41467-022-34009-x (PMC9586930; doi:10.1038/s41467-022-34009-x)
Supplement: Supplementary file 3 — Description of Additional Supplementary Files [file 41467_2022_34009_MOESM3_ESM.pdf]

Description of Additional Supplementary Files

File Name: Supplementary Movie 1
Description: 3D variability analysis of ABL–bound PTH1R–Gs complex, related to Figures 2.

File Name: Supplementary Movie 2
Description: 3D variability analysis of PTH–bound PTH1R–Gs complex, related to Figures 2.

File Name: Supplementary Movie 3
Description: 3D variability analysis of LA-PTH–bound PTH1R–Gs complex, related to Figures 2.
