# Supplementary material for: Molecular insights into the distinct signaling duration for the peptide-induced PTH1R activation
Source: Nat Commun. 2022 Oct 21;13:6276. doi: 10.1038/s41467-022-34009-x (PMC9586930; doi:10.1038/s41467-022-34009-x)
Supplement: Supplementary file 5 — Reporting Summary [file 41467_2022_34009_MOESM5_ESM.pdf]

Corresponding author(s): Zhihong Liu, Yan Zhang, Chunyou Mao

Last updated by author(s): Sep 25, 2022

Reporting Summary

Nature Portfolio wishes to improve the reproducibility of the work that we publish. This form provides structure for consistency and transparency in reporting. For further information on Nature Portfolio policies, see our Editorial Policies and the Editorial Policy Checklist.

Statistics

For all statistical analyses, confirm that the following items are present in the figure legend, table legend, main text, or Methods section.

n/a	Confirmed	
☐	☒	The exact sample size (*n*) for each experimental group/condition, given as a discrete number and unit of measurement
☐	☒	A statement on whether measurements were taken from distinct samples or whether the same sample was measured repeatedly
☐	☒	The statistical test(s) used AND whether they are one- or two-sided<br>*Only common tests should be described solely by name; describe more complex techniques in the Methods section.*
☒	☐	A description of all covariates tested
☒	☐	A description of any assumptions or corrections, such as tests of normality and adjustment for multiple comparisons
☐	☒	A full description of the statistical parameters including central tendency (e.g. means) or other basic estimates (e.g. regression coefficient) AND variation (e.g. standard deviation) or associated estimates of uncertainty (e.g. confidence intervals)
☐	☒	For null hypothesis testing, the test statistic (e.g. *F*, *t*, *r*) with confidence intervals, effect sizes, degrees of freedom and *P* value noted<br>*Give P values as exact values whenever suitable.*
☒	☐	For Bayesian analysis, information on the choice of priors and Markov chain Monte Carlo settings
☒	☐	For hierarchical and complex designs, identification of the appropriate level for tests and full reporting of outcomes
☒	☐	Estimates of effect sizes (e.g. Cohen's *d*, Pearson's *r*), indicating how they were calculated

Our web collection on statistics for biologists contains articles on many of the points above.

Software and code

Policy information about availability of computer code

Data collection	Cryo-EM data collection was performed using SerialEM3.8.
Data analysis	The following software was used in this study: MotionCor2.1, Gctfl.18, RELION 3.1, CryoSPARC v3.2, Bsoft 2.0.7, Rosetta 2019.35, DeepEMhancer 1.0, Coot 0.8.9, Phenix 1.18, UCSF Chimera 1.14, UCSF ChimeraX 1.4, Graphpad Prism 8.

For manuscripts utilizing custom algorithms or software that are central to the research but not yet described in published literature, software must be made available to editors and reviewers. We strongly encourage code deposition in a community repository (e.g. GitHub). See the Nature Portfolio guidelines for submitting code & software for further information.

Data

Policy information about availability of data

All manuscripts must include a data availability statement. This statement should provide the following information, where applicable:

- Accession codes, unique identifiers, or web links for publicly available datasets
- A description of any restrictions on data availability
- For clinical datasets or third party data, please ensure that the statement adheres to our policy

The atomic coordinates and the electron microscopy maps of ABL– and PTH–bound PTH1R–Gs complexes have been deposited in the Protein Data Bank (PDB) under accession numbers 7Y35, 7Y36 and Electron Microscopy Data Bank (EMDB) under accession codes 33588, 33590 respectively. The structural data used in this study have been deposited in the PDB under accession number 6NBF for LA-PTH–PTH1R–Gs complex, 6FJ3 for ePTH–PTH1R complex, 6LMK for Glucagon–GCGR–Gs complex, 5XEZ for inactive GCGR, 6VCB for GLP-1GLP-1R–Gs complex, 6LN2 for inactive GLP-1R, 5YQZ for Glucagon analogue–GCGR complex, 7F16 for TIP39–

PTH2R–Gs complex. All other data generated in this study are provided in the Supplementary information, Supplementary files and source data files.

Human research participants

Policy information about studies involving human research participants and Sex and Gender in Research.

Reporting on sex and gender	N/A
Population characteristics	N/A
Recruitment	N/A
Ethics oversight	N/A

Note that full information on the approval of the study protocol must also be provided in the manuscript.

Field-specific reporting

Please select the one below that is the best fit for your research. If you are not sure, read the appropriate sections before making your selection.

- [x] Life sciences
- [ ] Behavioural & social sciences
- [ ] Ecological, evolutionary & environmental sciences

For a reference copy of the document with all sections, see nature.com/documents/nr-reporting-summary-flat.pdf

Life sciences study design

All studies must disclose on these points even when the disclosure is negative.

Sample size	Sample sizes were not predetermined by statistical methods. For cryo-EM data, sample sizes were determined by availability of microscope.Cryo-EM data was collected until we were able to define a high-resolution structure that allowed us to obtain a high-resolution reconstruction within the confines of limited microscope time.
Data exclusions	No data was excluded from the analyses. The procedure of generating 3D maps from cryo-EM particles involves sorting of particles that are damaged or are false-picked that are unlikely to refine correctly.This is implemented in RELION 3.1
Replication	Our primary data is cryo-EM structure that was calculated according to standard procedures and does not need replicates. The biochemical experiments in this study have been repeated by greater than or equal to three independent experiments, and those finding are reliably reproduced.
Randomization	Randomization is not relevant to this study, as protein and our experiments did not involve choosing. Our structure was calculated according to standard procedures with freely available software and does not need randomization
Blinding	Our experiments were all biochemical studies, no blinding was used or necessary during data collection or analysis. As above, Our primary data is a cryo-EM structure that was calculated according to standard procedures with freely available software and did not require blinding.

Reporting for specific materials, systems and methods

We require information from authors about some types of materials, experimental systems and methods used in many studies. Here, indicate whether each material, system or method listed is relevant to your study. If you are not sure if a list item applies to your research, read the appropriate section before selecting a response.

Materials & experimental systems

n/a	Involved in the study	
[ ]	[x]	Antibodies
[ ]	[x]	Eukaryotic cell lines
[x]	[ ]	Palaeontology and archaeology
[x]	[ ]	Animals and other organisms
[x]	[ ]	Clinical data
[x]	[ ]	Dual use research of concern

Methods

n/a	Involved in the study	
[x]	[ ]	ChIP-seq
[x]	[ ]	Flow cytometry
[x]	[ ]	MRI-based neuroimaging

Antibodies

Antibodies used	Antibodies used: anti-FLAG M2 HRP-conjugated monoclonal antibody (Sigma-Aldrich, Catalog Number A8592, Mouse IgG1)
Validation	The Anti-FLAG M2 HRP-conjugated monoclonal antibody is well characterized and was applied according to data sheet information details.<br>https://www.sigmaaldrich.cn/CN/zh/product/sigma/a8592

Eukaryotic cell lines

Policy information about cell lines and Sex and Gender in Research

Cell line source(s)	Sf9 cells, Expression systems, Cat. 94011S<br>HEK293 cells, ATCC, CRL-1573
Authentication	All of the cell lines are maintained by the supplier. No additional authentication was performed by the authors of this study
Mycoplasma contamination	The cell lines used in this study were negative for mycoplasma contamination
Commonly misidentified lines<br>(See ICLAC register)	No commonly misidentified cell lines were used
