# Supplementary material for: Molecular insights into the distinct signaling duration for the peptide-induced PTH1R activation
Source: Nat Commun. 2022 Oct 21;13:6276. doi: 10.1038/s41467-022-34009-x (PMC9586930; doi:10.1038/s41467-022-34009-x)
Supplement: Supplementary file 6 — Source Data [file 41467_2022_34009_MOESM6_ESM.zip › source data/source data-figure.docx]

**Source data Figure 1**


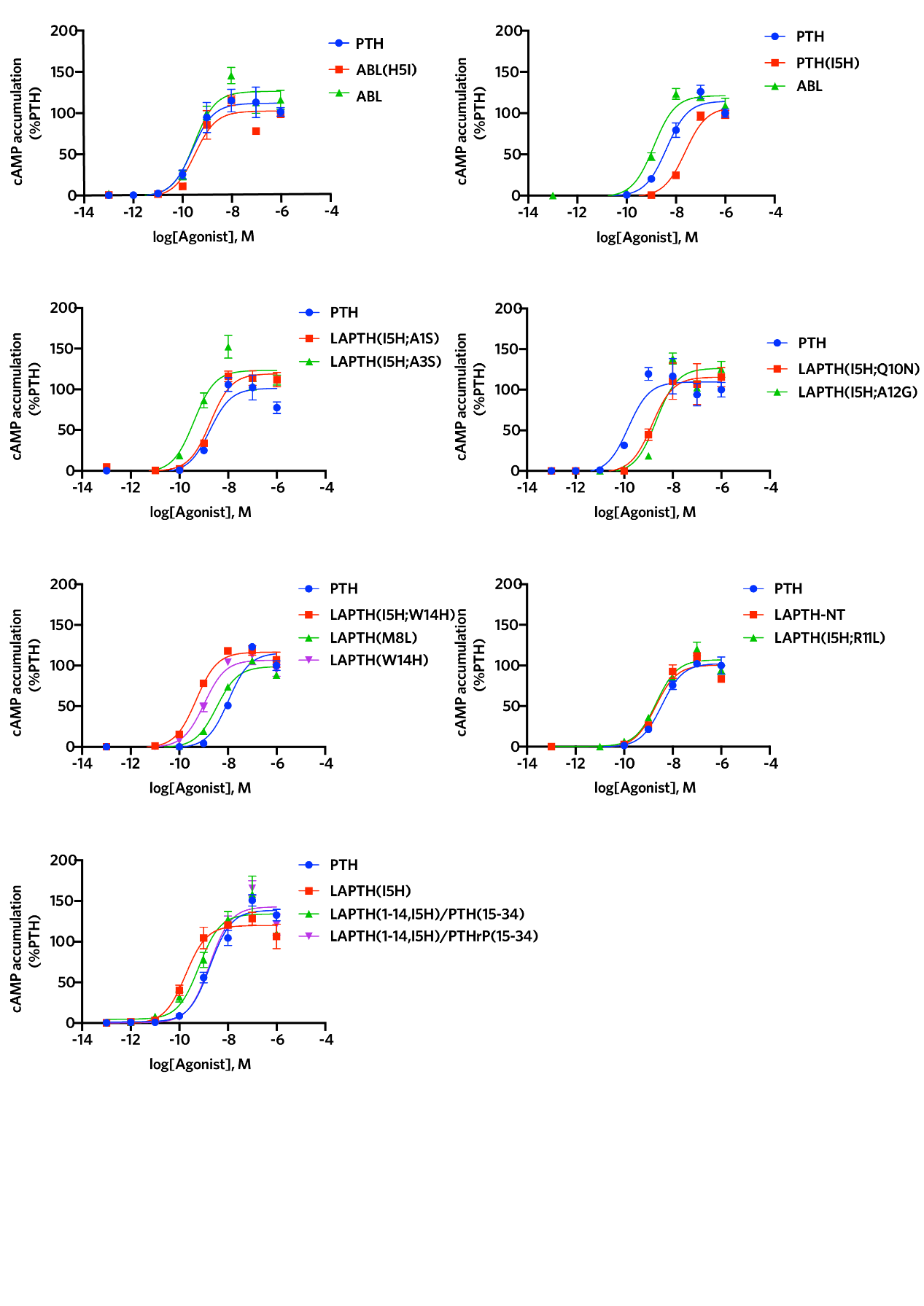


**Source data Figure.1** | cAMP accumulation assays of the peptide ligands used in this study.

Dose-response curves of cAMP accumulation were measured by Glosensor assay. The data were analyzed using the ‘log(agonist) vs. response-Variable slope (four parameters)’ function in Graphpad Prism. All data are presented as mean values ± standard error of measurement (SEM).

**Source data Table.1 | Sequence of peptides**

| **Name** | **Sequence** |
| --- | --- |
| PTH | H-SVSEIQLMHNLGKHLNSMERVEWLRKKLQDVHNF-OH |
| PTHrP(1-36) | H-AVSEHQLLHDKGKSIQDLRRRFFLHHLIAEIHTAEI-NH2 |
| ABL | H-AVSEHQLLHDKGKSIQDLRRRELLEKLL-{Aib}-KLHTA-NH2 |
| LA-PTH | H-AVAEIQLMHQRAKWIQDARRRAFLHKLIAEIHTAEI-COOH |
| I5H-LA-PTH | H-AVAEHQLMHQRAKWIQDARRRAFLHKLIAEIHTAEI |
| LA-PTH (I5H-PTHrP_CT) | H-AVAEHQLMHQRAKWIQDLRRRFFLHHLIAEIHTAEI-NH2 |
| LA-PTH (I5H-PTH_CT) | H-AVAEHQLMHQRAKWLNSMERVEWLRKKLQDVHNF-OH |
| LA-PTH (I5H-PTH_NT) | H-SVSEHQLMHNLGKHIQDARRRAFLHKLIAEIHTAEI-COOH |
| LA-PTH (I5H-PTH_1) | H-SVAEHQLMHQRAKWIQDARRRAFLHKLIAEIHTAEI-COOH |
| LA-PTH (I5H-PTH_3) | H-AVSEHQLMHQRAKWIQDARRRAFLHKLIAEIHTAEI-COOH |
| LA-PTH (I5H-PTH_10) | H-AVAEHQLMHNRAKWIQDARRRAFLHKLIAEIHTAEI-COOH |
| LA-PTH (I5H-PTH_11) | H-AVAEHQLMHQLAKWIQDARRRAFLHKLIAEIHTAEI-COOH |
| LA-PTH (I5H-PTH_12) | H-AVAEHQLMHQRGKWIQDARRRAFLHKLIAEIHTAEI-COOH |
| LA-PTH (I5H-PTH_14) | H-AVAEHQLMHQRAKHIQDARRRAFLHKLIAEIHTAEI-COOH |
| I5H-LA-PTH | H-AVAEHQLMHQRAKWIQDARRRAFLHKLIAEIHTAEI |
| M8L-LA-PTH | H-AVAEIQLLHQRAKWIQDARRRAFLHKLIAEIHTAEI |
| W14S-LA-PTH | H-AVAEIQLMHQRAKSIQDARRRAFLHKLIAEIHTAEI |
| H51-ABL | H-AVSEIQLLHDKGKSIQDLRRRELLEKLL-{Aib}-KLHTA-NH2 |
| I5H-TER | H-SVSEHQLMHNLGKHLNSMERVEWLRKKLQDVHNF-OH |

**Source data Table .2 | cAMP dose-response analyses in HEK293T cells^a^**

|  | pEC50 | Emax |
| --- | --- | --- |
| PTH | -8.448 | 103.1 |
| LAPTH(I5H) | -9.738 | 120.8 |
| LAPTH(1-14,I5H)/PTHrP(15-34) | -8.755 | 142.8 |
| LAPTH(1-14,I5H)/PTH(15-34) | -9.192 | 129.6 |
| LA-PTH (I5H-PTH;NT) | -8.703 | 102.1 |
| LAPTH(I5H;A1S) | -8.735 | 119.8 |
| LAPTH(I5H;A3S) | -9.409 | 125.3 |
| LAPTH(I5H;Q10N) | -8.854 | 117.7 |
| LAPTH(I5H;R11L) | -8.682 | 106.9 |
| LAPTH(I5H;A12G) | -8.681 | 129.5 |
| LAPTH(I5H;W14H) | -9.307 | 117.8 |
| LAPTH(I5H) | -9.738 | 120.8 |
| LAPTH(M8L) | -8.462 | 99.71 |
| LAPTH(W14H) | -8.974 | 107.5 |
| ABL(H5I) | -9.495 | 104.1 |
| PTH(I5H) | -7.628 | 108.9 |

a, Assays were performed in HEK293T cells; values of half-maximal stimulatory concentration (as pEC50) and Emax (as luminescence counts per 500ms) were derived from curve fitting dose-response data.
