# Supplementary material for: Molecular insights into the distinct signaling duration for the peptide-induced PTH1R activation
Source: Nat Commun. 2022 Oct 21;13:6276. doi: 10.1038/s41467-022-34009-x (PMC9586930; doi:10.1038/s41467-022-34009-x)
Supplement: Supplementary file 6 — Source Data [file 41467_2022_34009_MOESM6_ESM.zip › source data/biophysical analyses and purity assessment/ABL-HPLC.pdf]

peptide
Professional R&D Partner

CERTIFICATE OF ANALYSIS

**Product Name**	Abaloparatide
**Lot No**	JT-94027
**Sequence**	H-AVSEHQLLHDKGKSIQDLRRRELLEKLL-{Aib}-KLHTA-NH2
**Dissolution condition**	15%ACN+85%H2O
**Length**	34AA
**Modification**	N/A
**Molecular Weight (MW)**	3960.58
**Storage**	-20℃

**Test Items**	**Specifications**	**Results**
**Purity by HPLC**	95%	95.58%
**Peptide Content**	N/A	N/A
**Moisture content**	N/A	N/A
**Acetic acid content**	N/A	N/A
**Appearance**	White to off-white lyophilized powder	Conforms
**Quantity**	500mg	10mg*50

Certified by:
Quality Assurance Department

Date 02-03-2021

Note: this product is intended for research use only; not for diagnostic or human use.

Web:www.anlianpeptide.com
Address:Dongbao Building, Songjiang District, Shanghai
Tel:+86-18521331115

Sample Information

Order ID	: Syn-94027
Name	: Abaloparatide
Sequence	: H-AVSEHQLLHDKGKSIQDLRRRELLEKLL-{Aib}-KLHTA-NH2
Lot No	: JT-94027
Pump A	: 0.1% Trifluoroacetic in 100% Water
Pump B	: 0.1% Trifluoroacetic in 100% Acetonrtrile
Total Flow	: 1ml/min
Wavelength	: 220nm
Analytial column type	: SHIMADZU Inertsil ODS-SP（4.6*250mm*5um）
Inj. Volume	: 30ul

Time	Module	Action	Value
0.00	Pumps	B.Conc	15
25.00	Pumps	B.Conc	75
25.01	Pumps	B.Conc	100
30.00	Pumps	B.Conc	100
30.01	Pumps	Stop	

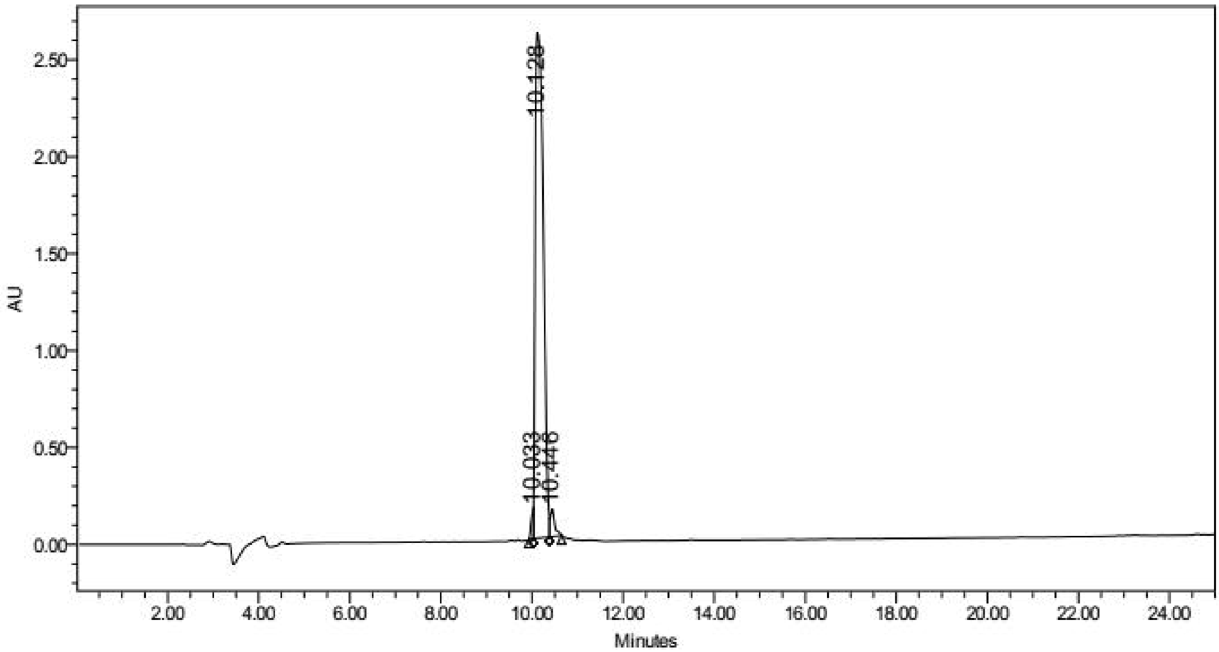

	RT	Area	% Area	Height
1	10.033	491000	1.47	157132
2	10.128	31848234	95.58	2652190
3	10.446	981195	2.94	140565

Web:www.anlianpeptide.com
Address:Dongbao Building, Songjiang District, Shanghai
Tel:+86-18521331115
