# Supplementary material for: Molecular insights into the distinct signaling duration for the peptide-induced PTH1R activation
Source: Nat Commun. 2022 Oct 21;13:6276. doi: 10.1038/s41467-022-34009-x (PMC9586930; doi:10.1038/s41467-022-34009-x)
Supplement: Supplementary file 6 — Source Data [file 41467_2022_34009_MOESM6_ESM.zip › source data/biophysical analyses and purity assessment/LAPTH(I5H)-HPLC.pdf]

CERTIFICATE OF ANALYSIS

**Product Name**	I5H-LA
**Lot No**	JT-99916
**Sequence**	AVAEHQLMHQRAKWIQDARRRAFLHKLIAEIHTAEI
**Dissolution condition**	15%ACN+85%H2O
**Length**	36AA
**Modification**	N/A
**Molecular Weight (MW)**	4288.96
**Storage**	-20℃

**Test Items**	**Specifications**	**Results**
**Purity by HPLC**	>95%	95.22%
**Peptide Content**	N/A	N/A
**Moisture content**	N/A	N/A
**Acetic acid content**	N/A	N/A
**Appearance**	White to off-white lyophilized powder	Conforms
**Quantity**	5.0mg	1.0mg*5

Certified by:
Quality Assurance Department

Date 11/10/2021

Note: this product is intended for research use only; not for diagnostic or human use.

Sample Information

Order ID : Syn-99916
Name : I5H-LA
Sequence : AVAEHQLMHQRAKWIQDARRRAFLHKLIAEIHTAEI
Lot No : JT-99916
Pump A : 0.1% Trifluoroacetic in 100% Water
Pump B : 0.1% Trifluoroacetic in 100% Acetonrtrile
Total Flow : 1ml/min
Wavelength : 220nm
Analytial column type : SHIMADZU Inertsil ODS-SP（4.6*250mm*5um）
Inj. Volume : 30ul

Time	Module	Action	Value
0.00	Pumps	B.Conc	25
25.00	Pumps	B.Conc	85
25.01	Pumps	B.Conc	100
30.00	Pumps	B.Conc	100
30.01	Pumps	Stop	

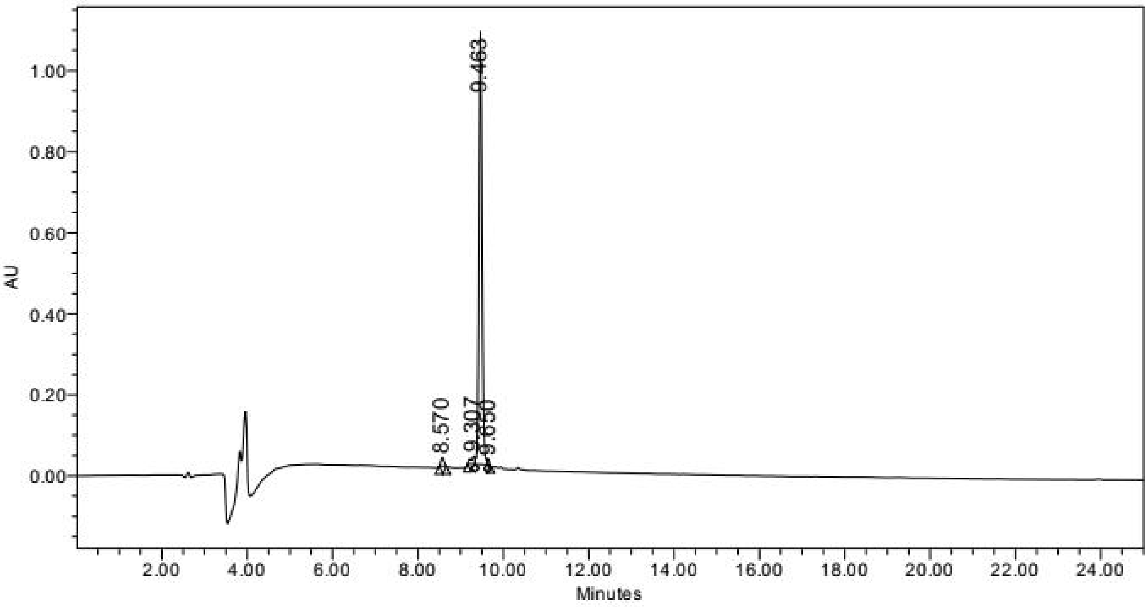

	RT	Area	% Area	Height
1	8.570	109109	1.91	21110
2	9.307	126425	2.22	19768
3	9.463	5425509	95.22	1059053
4	9.650	36804	0.65	13975

Address:Dongbao Building, Songjiang District, Shanghai
Tel:+86-18521331115
