# Supplementary material for: Molecular insights into the distinct signaling duration for the peptide-induced PTH1R activation
Source: Nat Commun. 2022 Oct 21;13:6276. doi: 10.1038/s41467-022-34009-x (PMC9586930; doi:10.1038/s41467-022-34009-x)
Supplement: Supplementary file 6 — Source Data [file 41467_2022_34009_MOESM6_ESM.zip › source data/biophysical analyses and purity assessment/PTH-HPLC.pdf]

CERTIFICATE OF ANALYSIS

**Product Name**	Teriparatide
**Lot No**	JT-92740
**Sequence**	H-SVSEIQLMHNLGKHLNSMERVEWLRKKLQDVHNF-OH
**Dissolution condition**	15%ACN+85%H2O
**Length**	34AA
**Modification**	N/A
**Molecular Weight (MW)**	4117.77
**Storage**	-20℃

**Test Items**	**Specifications**	**Results**
**Purity by HPLC**	95%	95.10%
**Peptide Content**	N/A	N/A
**Moisture content**	N/A	N/A
**Acetic acid content**	N/A	N/A
**Appearance**	White to off-white lyophilized powder	Conforms
**Quantity**	50mg	10mg*5

Certified by:
Quality Assurance Department

Date 12-07-2020

Note: this product is intended for research use only; not for diagnostic or human use.

Web:www.anlianpeptide.com
Address:Dongbao Building, Songjiang District, Shanghai
Tel:+86-18521331115

Sample Information

Order ID : Syn-92740
Name : Teriparatide
Sequence : H-SVSEIQLMHNLGKHLNSMERVEWLRKKLQDVHNF-OH
Lot No : JT-92740
Pump A : 0.1% Trifluoroacetic in 100% Water
Pump B : 0.1% Trifluoroacetic in 100% Acetonrtrile
Total Flow : 1ml/min
Wavelength : 220nm
Analytial column type : SHIMADZU Inertsil ODS-SP（4.6*250mm*5um）
Inj. Volume : 30ul

Time	Module	Action	Value
0.00	Pumps	B.Conc	15
25.00	Pumps	B.Conc	75
25.01	Pumps	B.Conc	100
30.00	Pumps	B.Conc	100
30.01	Pumps	Stop	

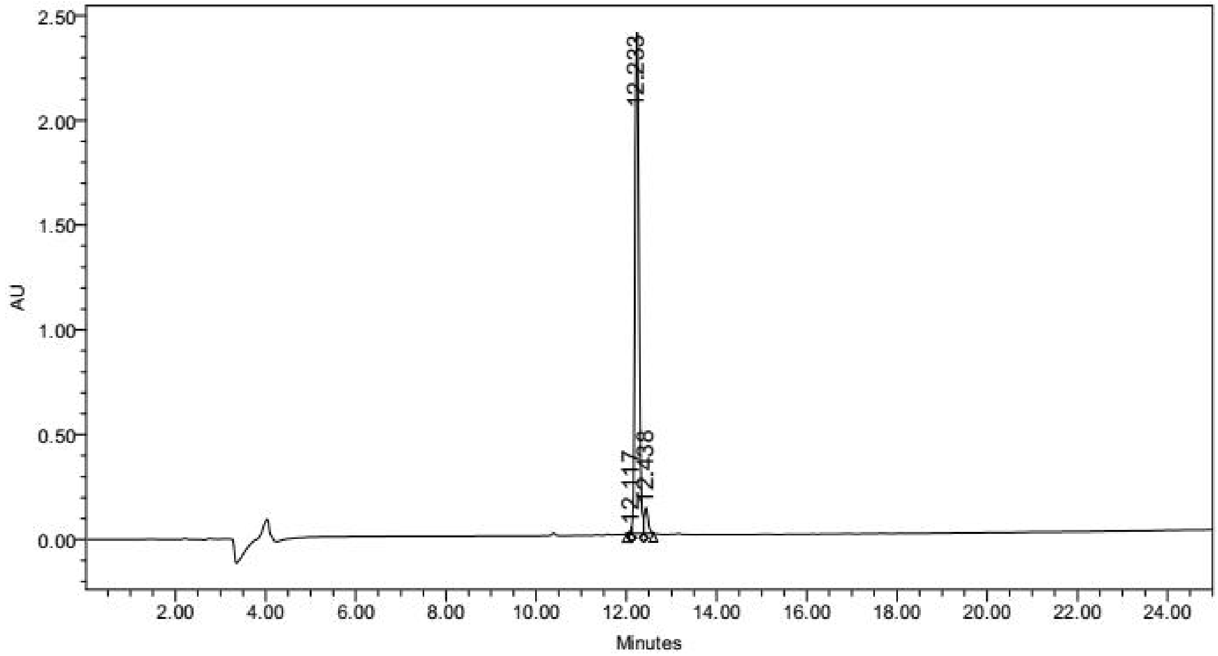

	RT	Area	% Area	Height
1	12.117	47180	0.30	25818
2	12.233	14746562	95.10	2390510
3	12.438	712924	4.60	119454

Web:www.anlianpeptide.com
Address:Dongbao Building, Songjiang District, Shanghai
Tel:+86-18521331115
