# Supplementary material for: Molecular insights into the distinct signaling duration for the peptide-induced PTH1R activation
Source: Nat Commun. 2022 Oct 21;13:6276. doi: 10.1038/s41467-022-34009-x (PMC9586930; doi:10.1038/s41467-022-34009-x)
Supplement: Supplementary file 6 — Source Data [file 41467_2022_34009_MOESM6_ESM.zip › source data/biophysical analyses and purity assessment/LAPTH(W14S)-HPLC.pdf]

Professional R&D Partner

CERTIFICATE OF ANALYSIS

**Product Name**	W14S-LA
**Lot No**	JT-99918
**Sequence**	AVAEIQLMHQRAKSIQDARRRAFLHKLIAEIHTAEI
**Dissolution condition**	15%ACN+85%H2O
**Length**	36AA
**Modification**	N/A
**Molecular Weight (MW)**	4165.84
**Storage**	-20℃

**Test Items**	**Specifications**	**Results**
**Purity by HPLC**	>95%	95.28%
**Peptide Content**	N/A	N/A
**Moisture content**	N/A	N/A
**Acetic acid content**	N/A	N/A
**Appearance**	White to off-white lyophilized powder	Conforms
**Quantity**	5.0mg	1.0mg*5

Certified by:
Quality Assurance Department

Date 11/15/2021

Note: this product is intended for research use only; not for diagnostic or human use.

Sample Information

Order ID	: Syn-99918
Name	: W14S-LA
Sequence	: AVAEIQLMHQRAKSIQDARRRAFLHKLIAEIHTAEI
Lot No	: JT-99918
Pump A	: 0.1% Trifluoroacetic in 100% Water
Pump B	: 0.1% Trifluoroacetic in 100% Acetonrtrile
Total Flow	: 1ml/min
Wavelength	: 220nm
Analytial column type	: SHIMADZU Inertsil ODS-SP（4.6*250mm*5um）
Inj. Volume	: 30ul

Time	Module	Action	Value
0.00	Pumps	B.Conc	20
25.00	Pumps	B.Conc	80
25.01	Pumps	B.Conc	100
30.00	Pumps	B.Conc	100
30.01	Pumps	Stop	

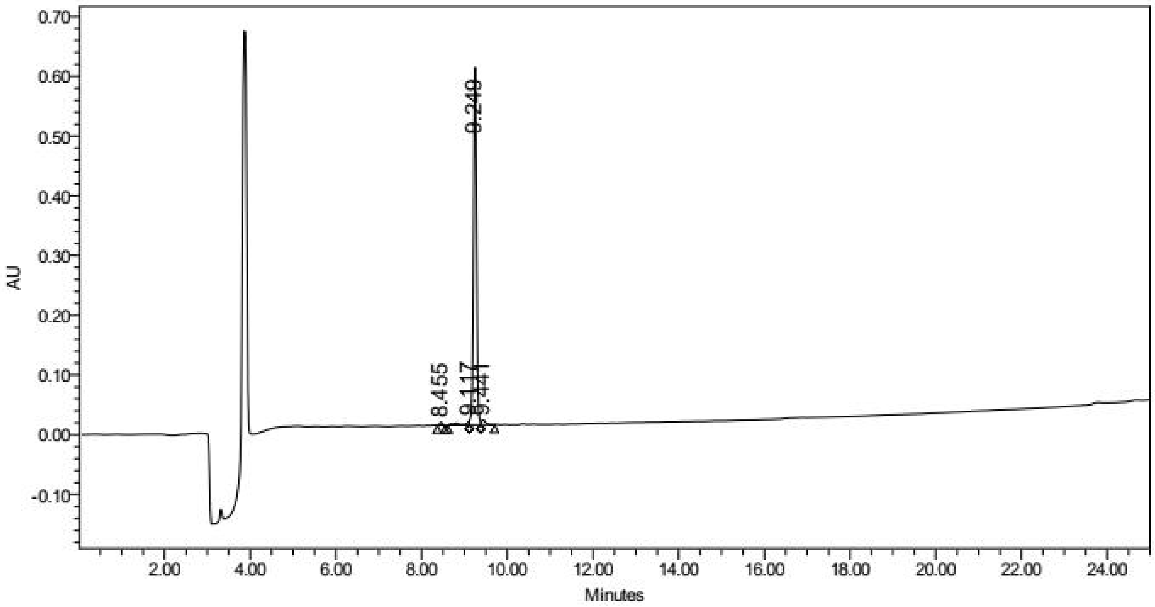

	RT	Area	% Area	Height
1	8.455	22442	0.75	4912
2	9.117	63311	2.11	7296
3	9.249	2855790	95.28	587425
4	9.441	55705	1.86	7779
