# Supplementary material for: Molecular insights into the distinct signaling duration for the peptide-induced PTH1R activation
Source: Nat Commun. 2022 Oct 21;13:6276. doi: 10.1038/s41467-022-34009-x (PMC9586930; doi:10.1038/s41467-022-34009-x)
Supplement: Supplementary file 6 — Source Data [file 41467_2022_34009_MOESM6_ESM.zip › source data/biophysical analyses and purity assessment/LA-PTH (I5H-PTH_CT)-HPLC.pdf]

CERTIFICATE OF ANALYSIS

**Product Name**	LA-PTH (I5H-PTH_CT)
**Lot No**	JT-162626
**Sequence**	AVAEHQLMHQRAKWLNSMERVEWLRKKLQDVHNF-COOH
**Dissolution condition**	15%ACN+85%H2O
**Length**	34AA
**Modification**	N/A
**Molecular Weight (MW)**	4229.86
**Storage**	-20℃

**Test Items**	**Specifications**	**Results**
**Purity by HPLC**	>95%	95.24%
**Peptide Content**	N/A	N/A
**Moisture content**	N/A	N/A
**Acetic acid content**	N/A	N/A
**Appearance**	White to off-white lyophilized powder	Conforms
**Quantity**	5.0mg	1.0mg*5

Certified by:
Quality Assurance Department

Date 03/08/2022

Note: this product is intended for research use only; not for diagnostic or human use.

Sample Information

Order ID : Syn-162626

Name : LA-PTH (I5H-PTH_CT)

Sequence : AVAEHQLMHQRAKWLNSMERVEWLRKKLQDVHNF-COOH

Lot No : JT-162626

Pump A : 0.1% Trifluoroacetic in 100% Water

Pump B : 0.1% Trifluoroacetic in 100% Acetonrtrile

Total Flow : 1ml/min

Wavelength : 220nm

Analytial column type : SHIMADZU Inertsil ODS-SP（4.6*250mm*5um）

Inj. Volume : 30ul

Time	Module	Action	Value
0.00	Pumps	B.Conc	20
25.00	Pumps	B.Conc	80
25.01	Pumps	B.Conc	100
30.00	Pumps	B.Conc	100
30.01	Pumps	Stop	

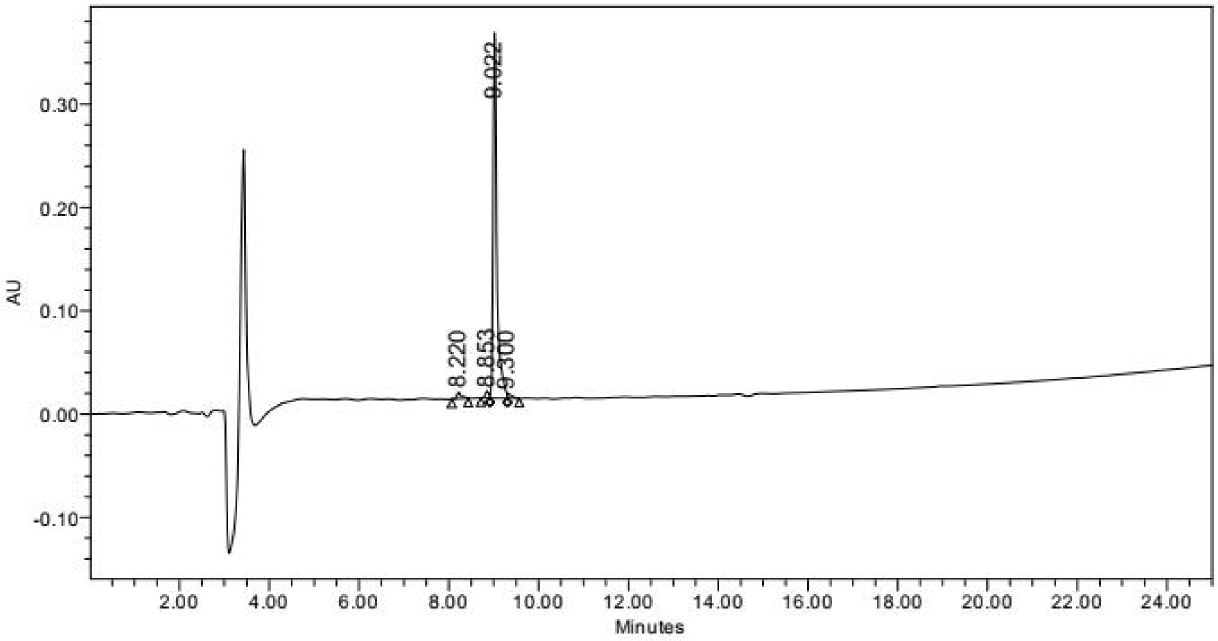

	RT	Area	% Area	Height
1	8.220	40682	1.88	5938
2	8.853	33897	1.56	6185
3	9.022	2064887	95.24	350490
4	9.300	28725	1.32	4845
