# Supplementary figures and images for: Molecular insights into the distinct signaling duration for the peptide-induced PTH1R activation
Source: Nat Commun. 2022 Oct 21;13:6276. doi: 10.1038/s41467-022-34009-x (PMC9586930; doi:10.1038/s41467-022-34009-x)

99917 P 6 (0.111)

Scan ES+  
6.85e7

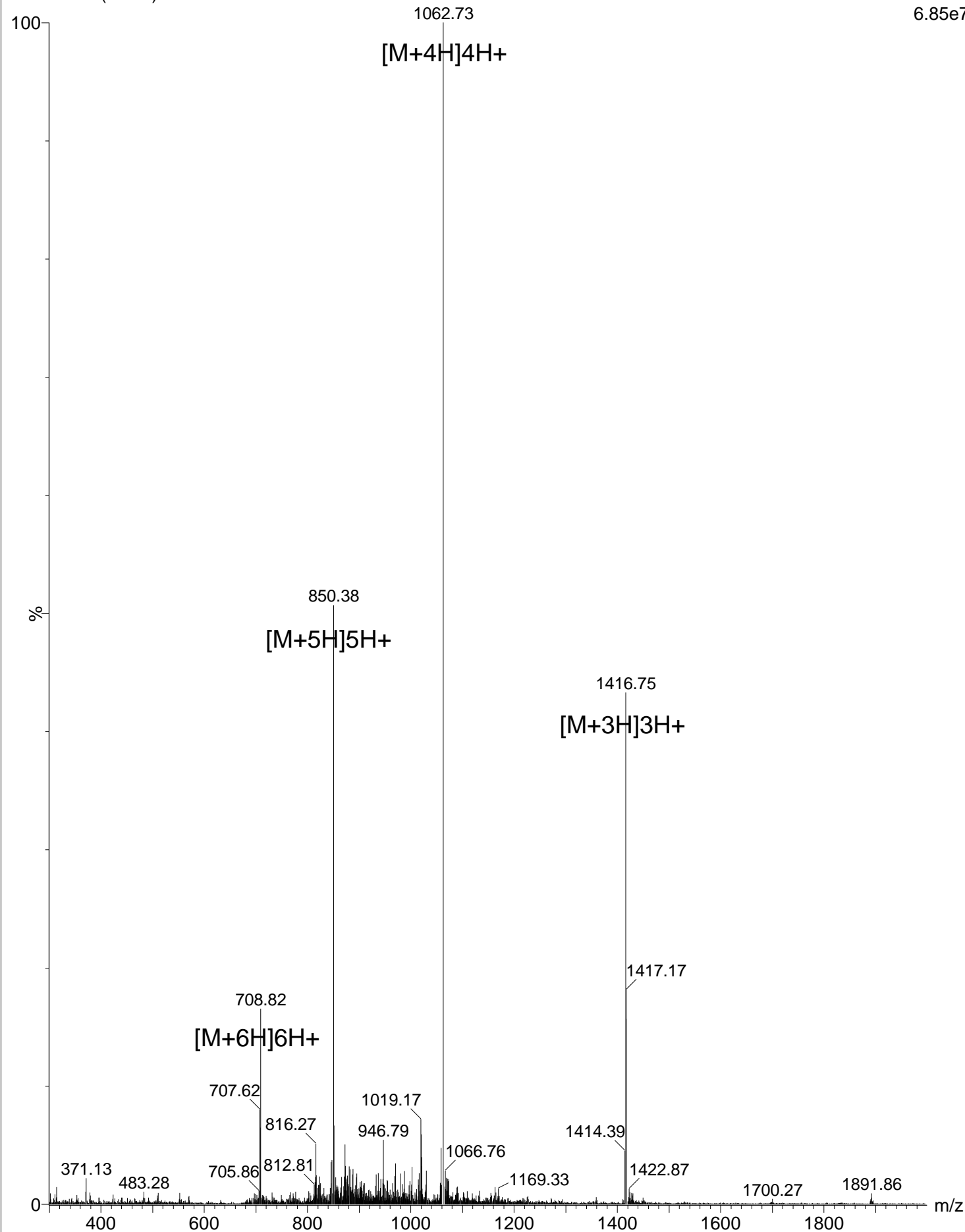

Supplement: Supplementary file 6 — Source Data [file 41467_2022_34009_MOESM6_ESM.zip › source data/biophysical analyses and purity assessment/LAPTH(M8L)-MS.pdf]

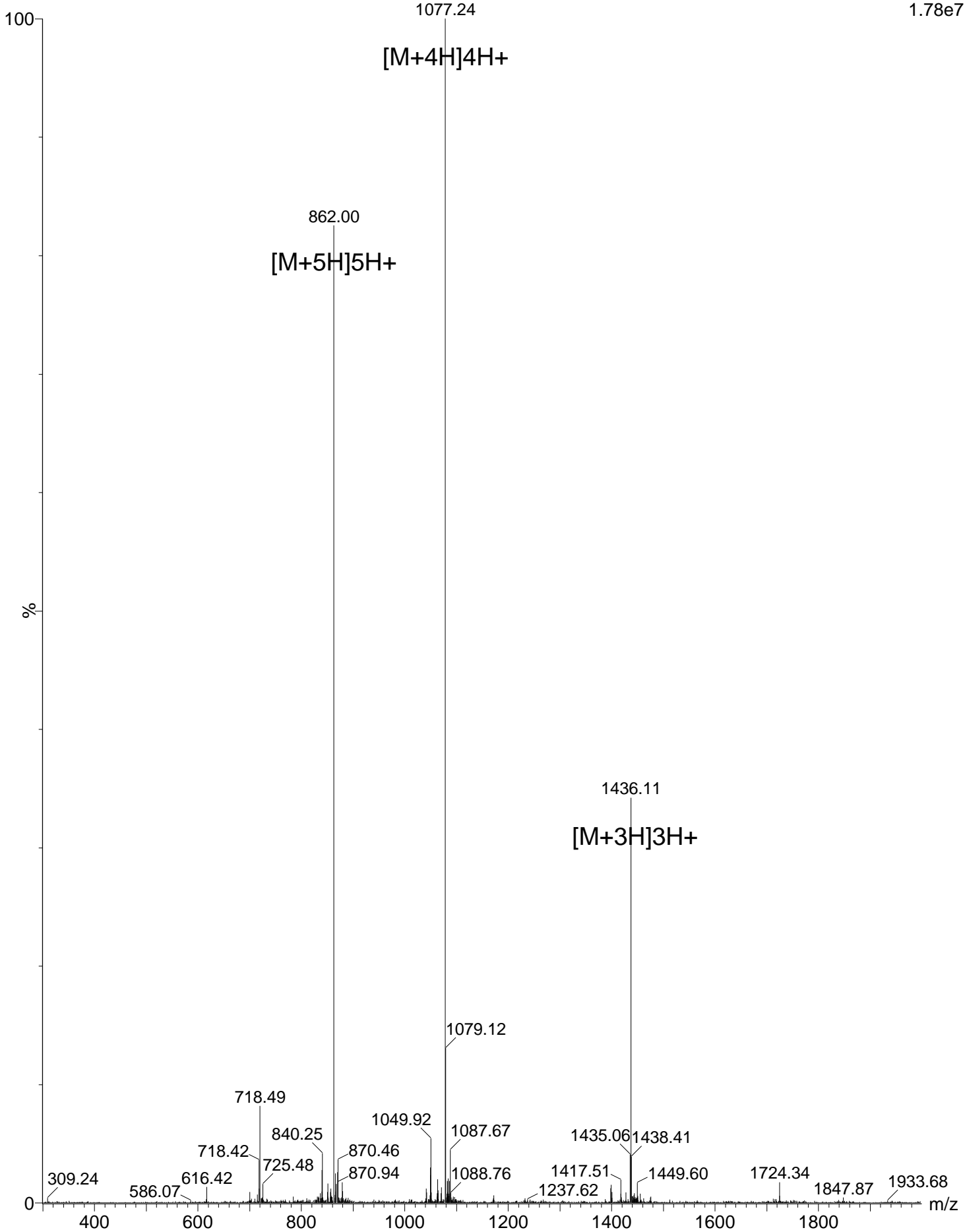

Supplement: Supplementary file 6 — Source Data [file 41467_2022_34009_MOESM6_ESM.zip › source data/biophysical analyses and purity assessment/LAPTH(I5H;A1S)-MS.pdf]

94027 P 6 (0.111)

Scan ES+  
2.91e7

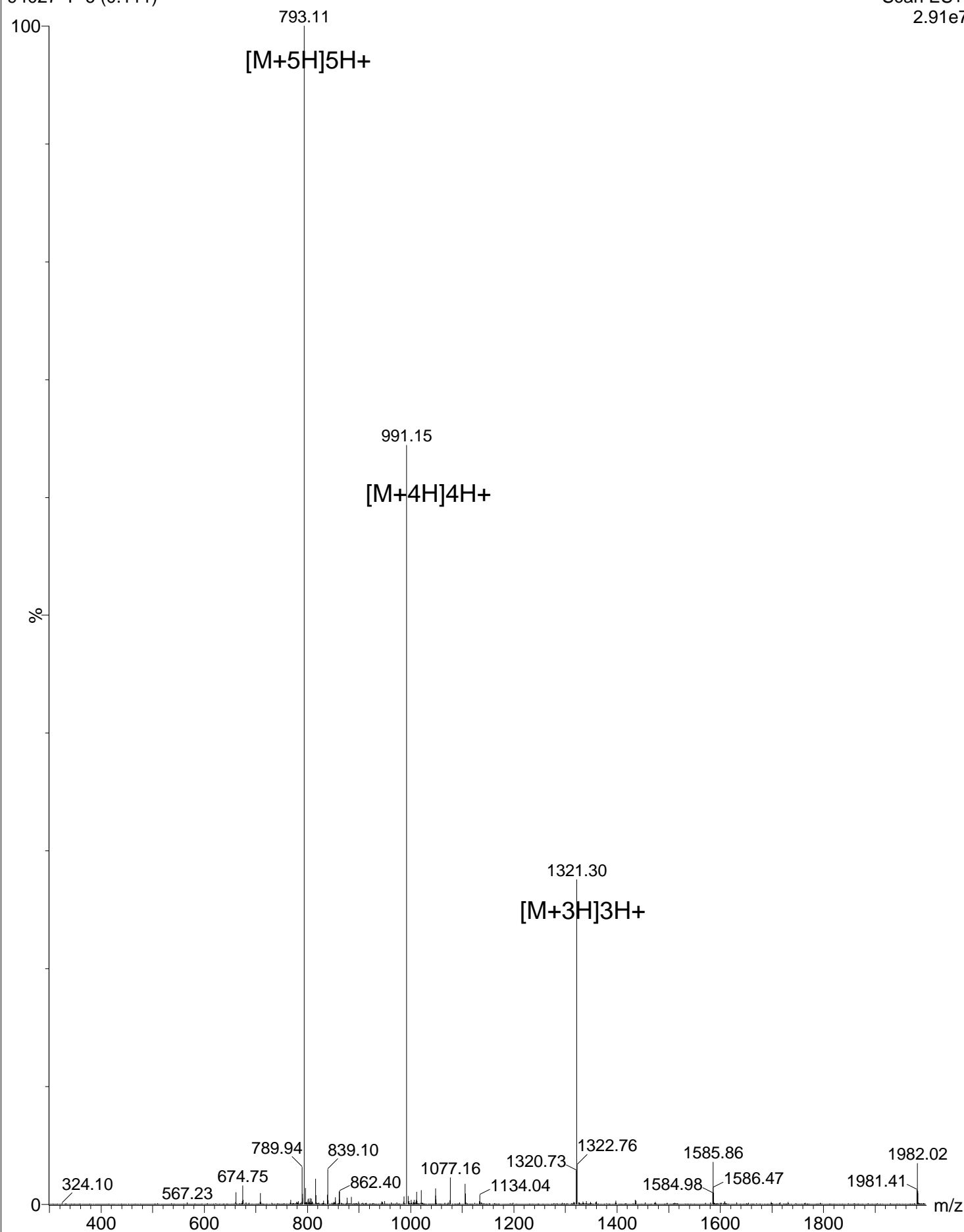

Supplement: Supplementary file 6 — Source Data [file 41467_2022_34009_MOESM6_ESM.zip › source data/biophysical analyses and purity assessment/ABL-MS.pdf]

162633 P 8 (0.148)

Scan ES+  
5.65e7

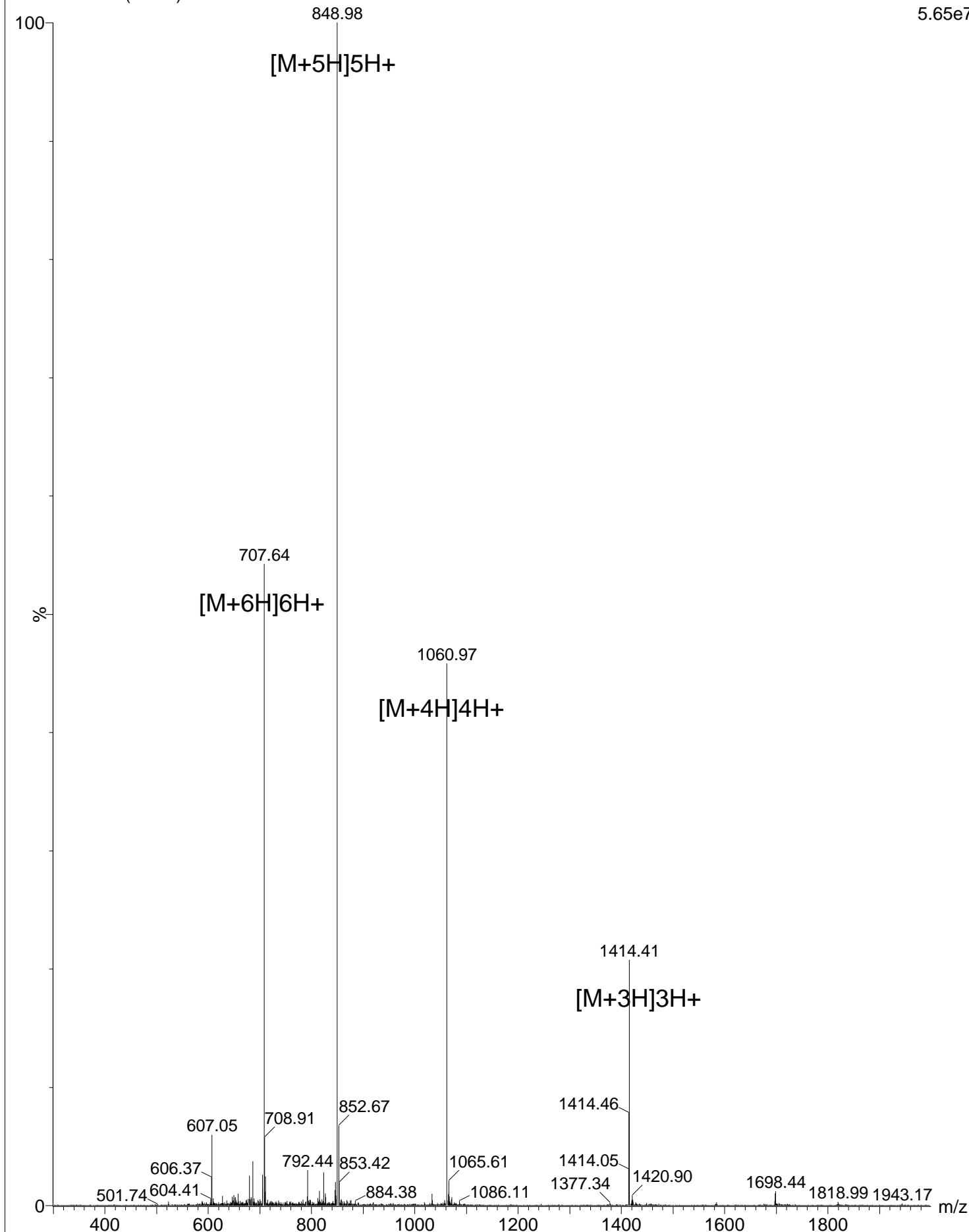

Supplement: Supplementary file 6 — Source Data [file 41467_2022_34009_MOESM6_ESM.zip › source data/biophysical analyses and purity assessment/LAPTH(I5H;W14H)-MS.pdf]

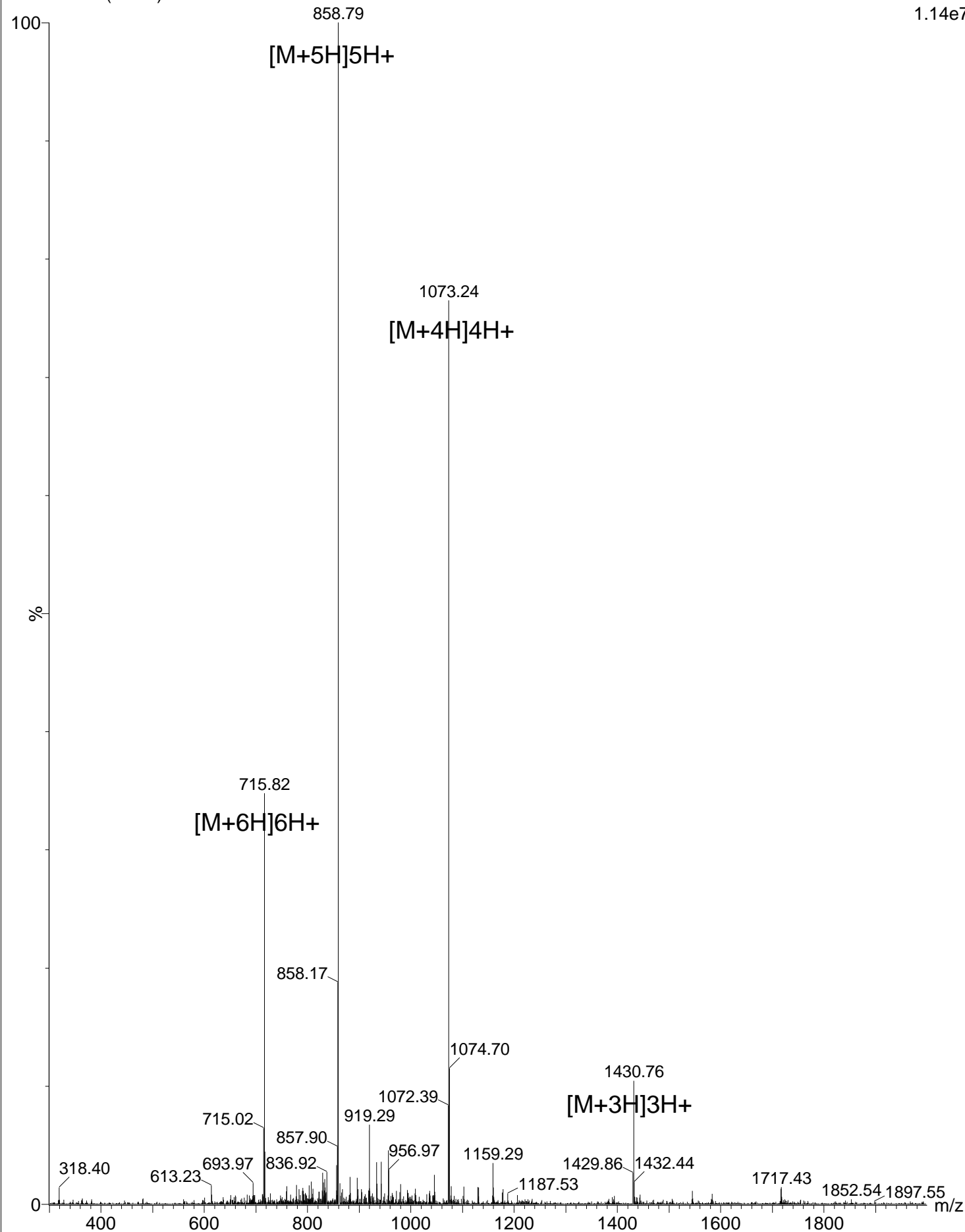

Supplement: Supplementary file 6 — Source Data [file 41467_2022_34009_MOESM6_ESM.zip › source data/biophysical analyses and purity assessment/LAPTH(I5H)-MS.pdf]

162626 P 3 (0.055)

Scan ES+  
4.06e7

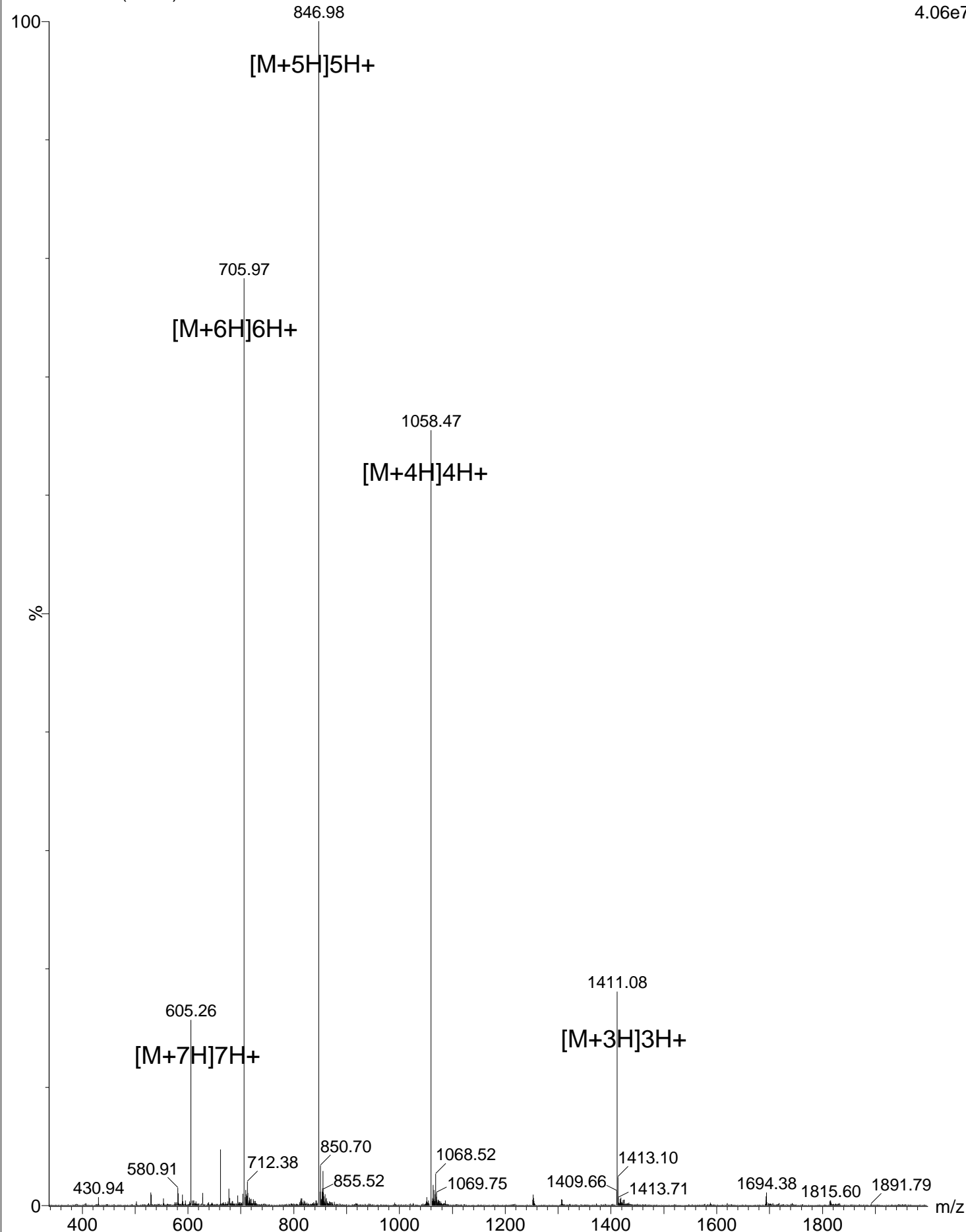

Supplement: Supplementary file 6 — Source Data [file 41467_2022_34009_MOESM6_ESM.zip › source data/biophysical analyses and purity assessment/LA-PTH (I5H-PTH_CT)-MS.pdf]

162631-2 P 17 (0.314)

Scan ES+  
3.94e6

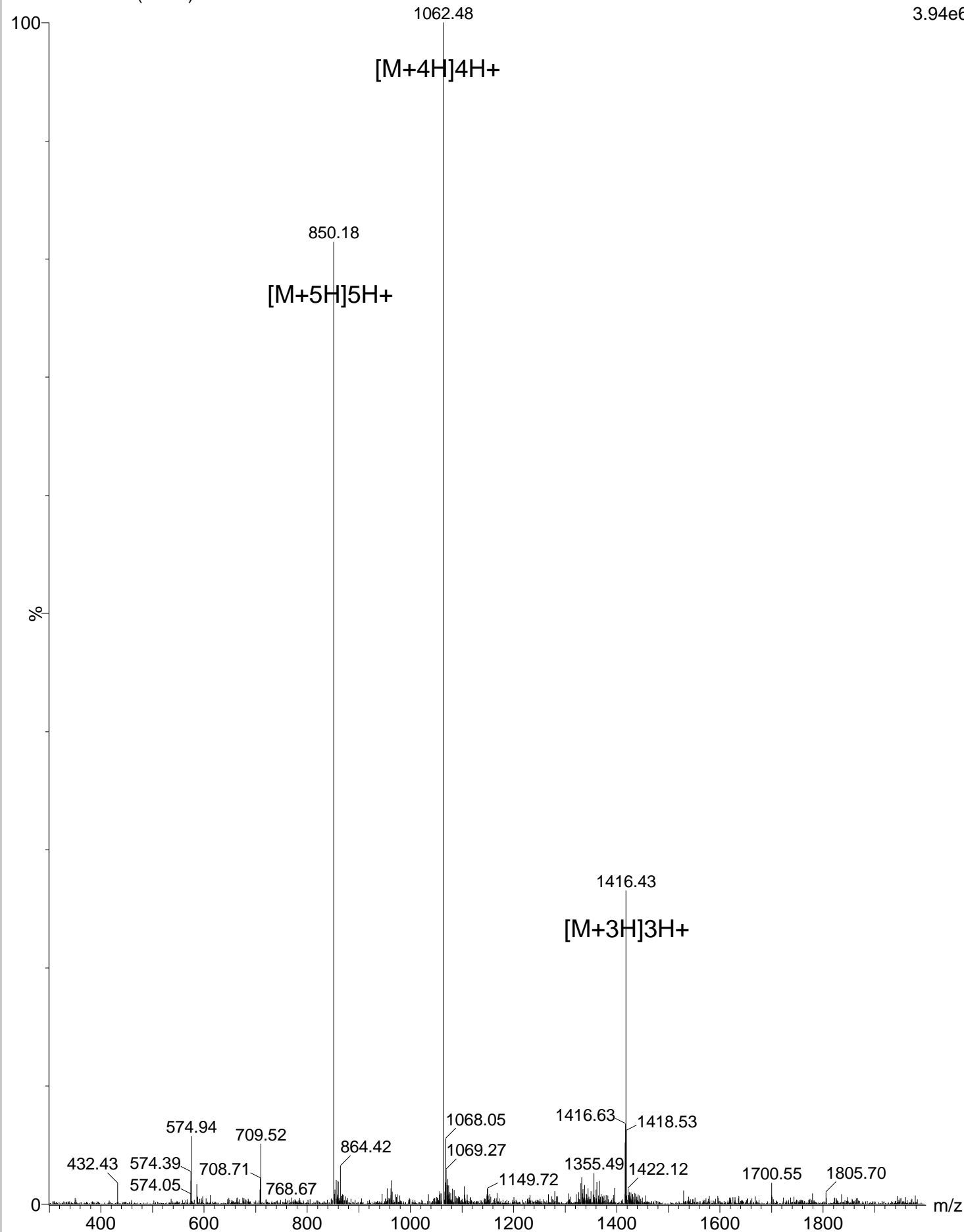

Supplement: Supplementary file 6 — Source Data [file 41467_2022_34009_MOESM6_ESM.zip › source data/biophysical analyses and purity assessment/LAPTH(I5H;R11L)-MS.pdf]

93300 P 5 (0.092)

Scan ES+  
2.67e7

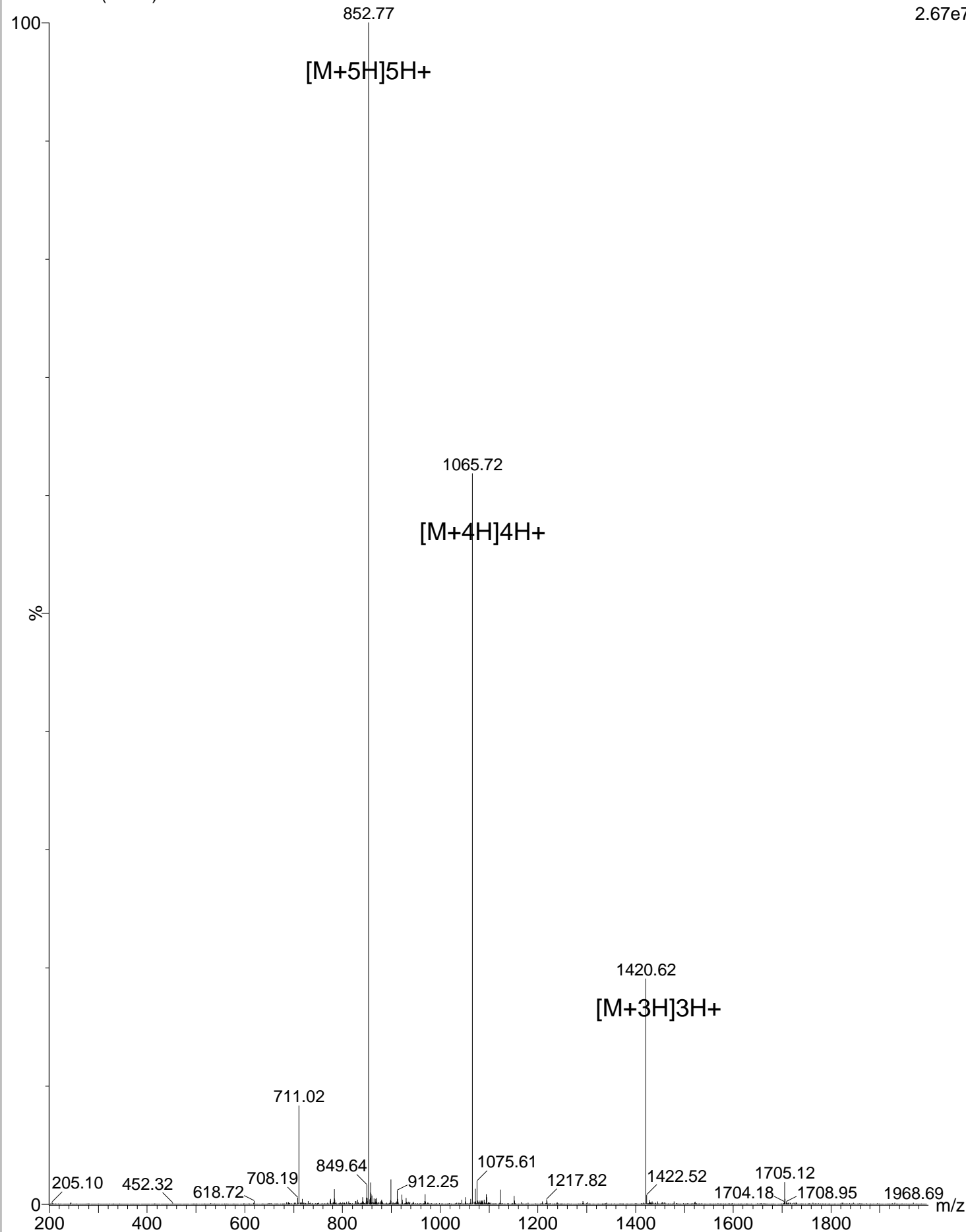

Supplement: Supplementary file 6 — Source Data [file 41467_2022_34009_MOESM6_ESM.zip › source data/biophysical analyses and purity assessment/PTHrP(1-36)-MS.pdf]

162627 P 6 (0.111)

Scan ES+  
1.15e7

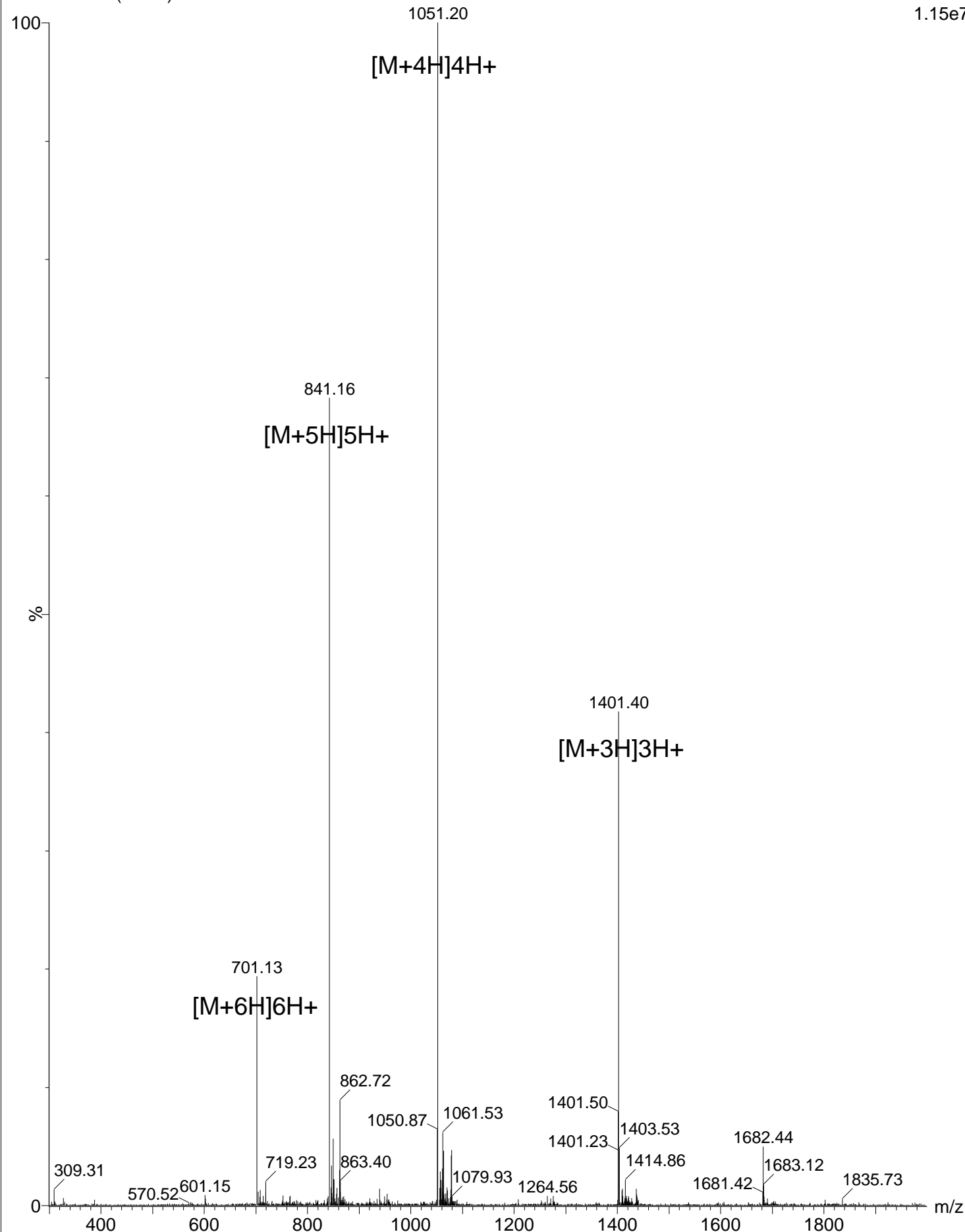

Supplement: Supplementary file 6 — Source Data [file 41467_2022_34009_MOESM6_ESM.zip › source data/biophysical analyses and purity assessment/LA-PTH (I5H-PTH_NT)-MS.pdf]

162625 P 4 (0.074)

Scan ES+  
3.35e7

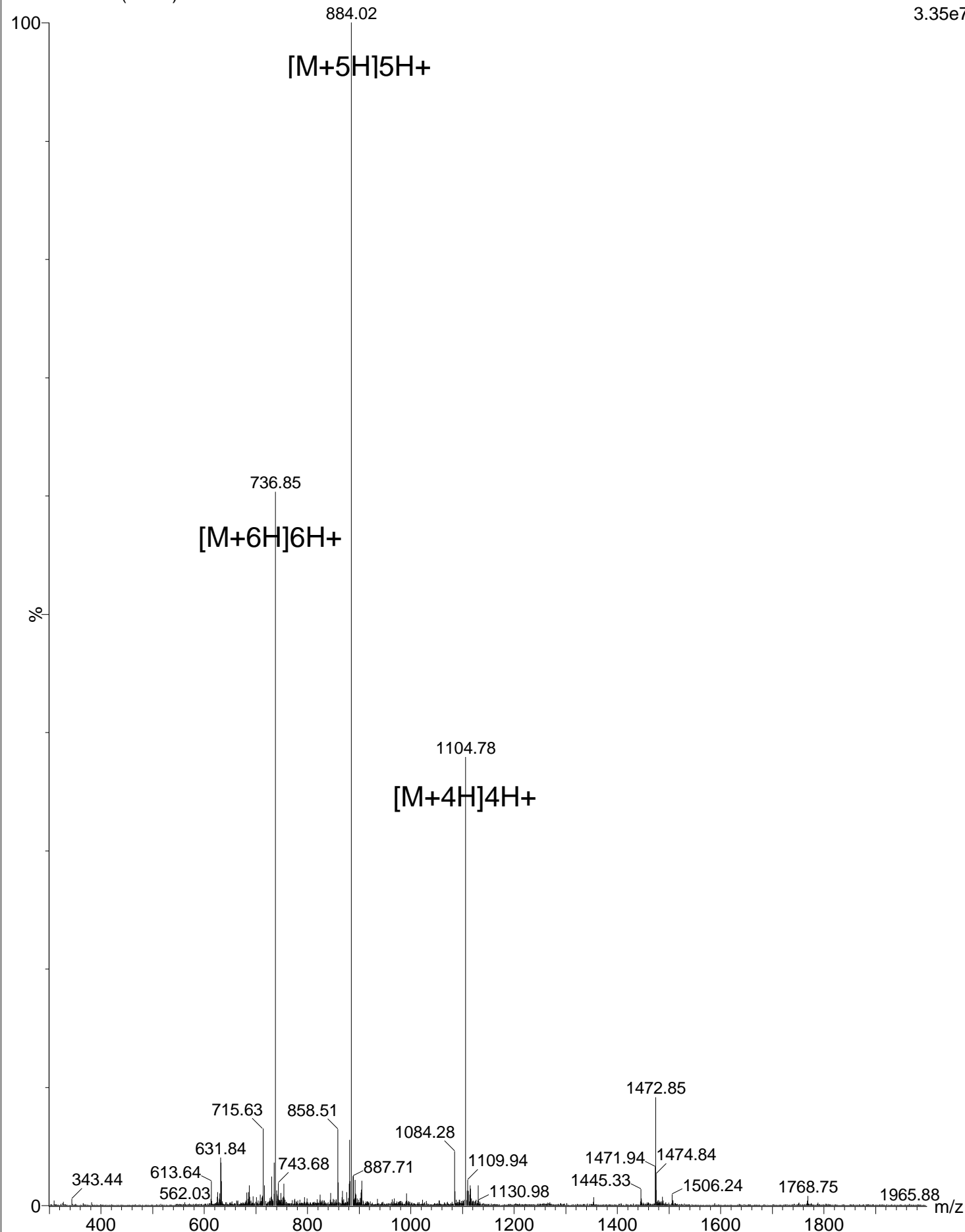

Supplement: Supplementary file 6 — Source Data [file 41467_2022_34009_MOESM6_ESM.zip › source data/biophysical analyses and purity assessment/LA-PTH (I5H-PTHrP_CT)-MS.pdf]

93809 P 6 (0.111)

Scan ES+  
2.93e7

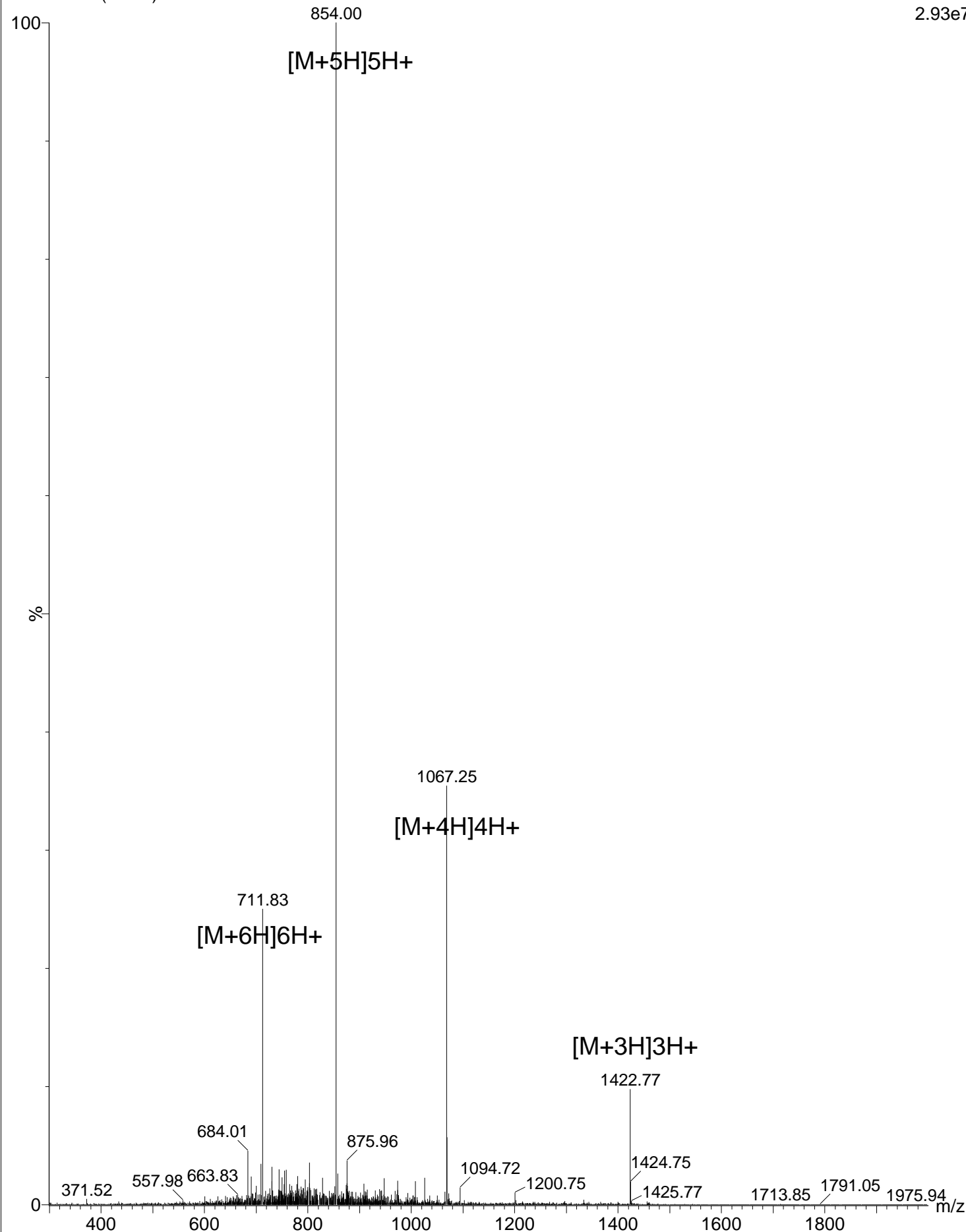

Supplement: Supplementary file 6 — Source Data [file 41467_2022_34009_MOESM6_ESM.zip › source data/biophysical analyses and purity assessment/LA-PTH-MS.pdf]

161856 P 5 (0.092)

Scan ES+  
2.05e7

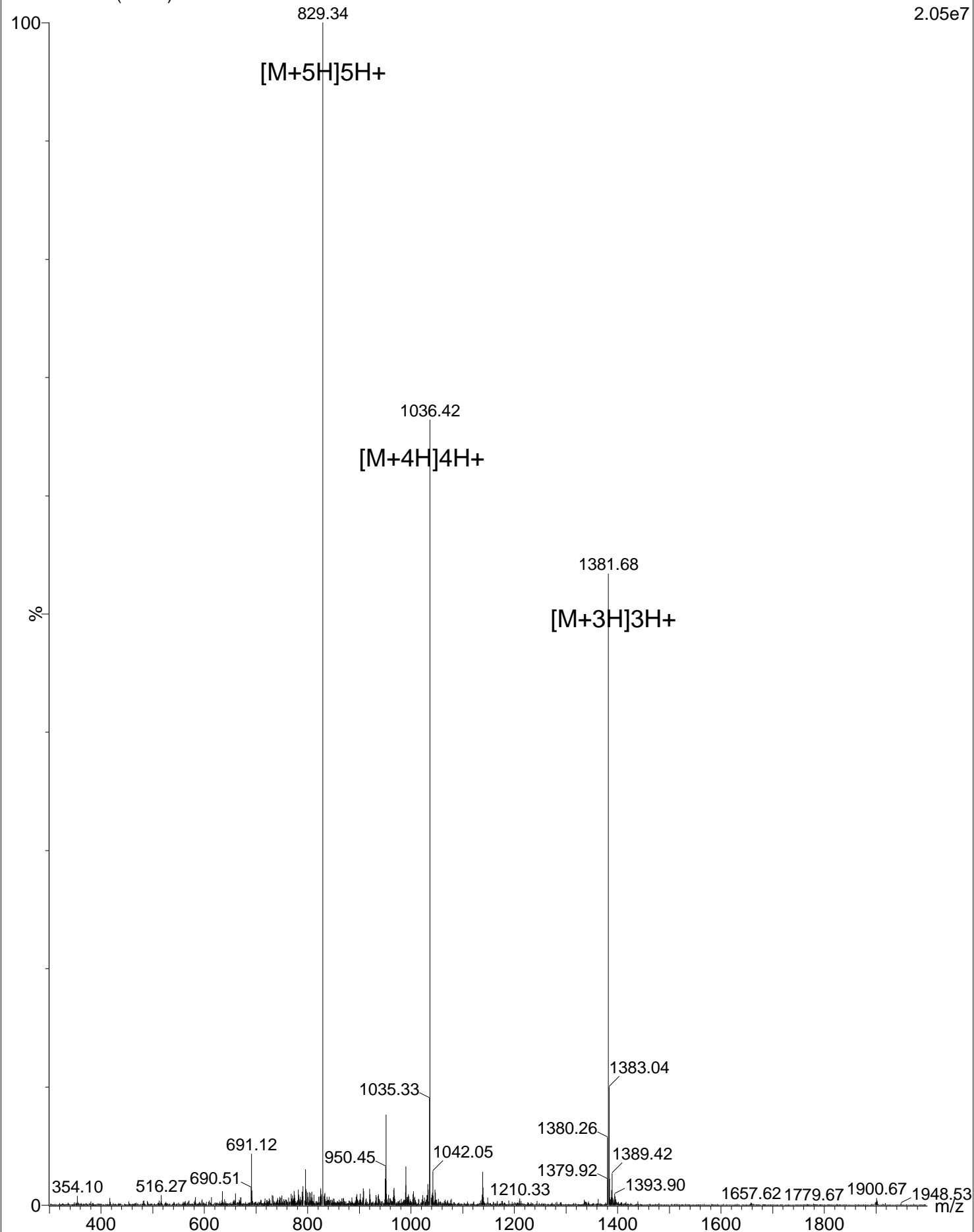

Supplement: Supplementary file 6 — Source Data [file 41467_2022_34009_MOESM6_ESM.zip › source data/biophysical analyses and purity assessment/PTH(I5H)-MS.pdf]

162630 P 5 (0.092)

Scan ES+  
7.76e7

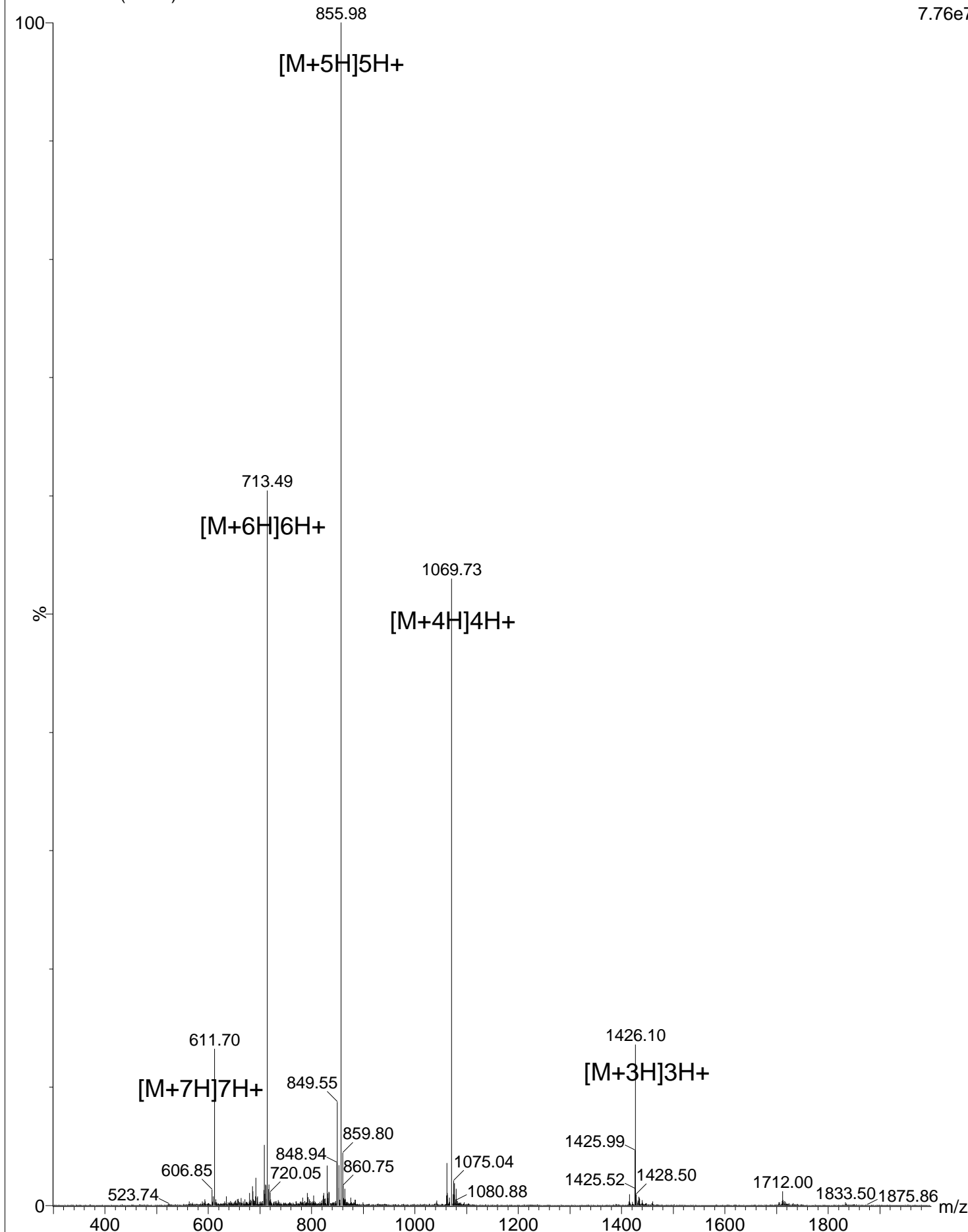

Supplement: Supplementary file 6 — Source Data [file 41467_2022_34009_MOESM6_ESM.zip › source data/biophysical analyses and purity assessment/LAPTH(I5H;Q10N)-MS.pdf]

162629 P 5 (0.092)

Scan ES+  
5.35e7

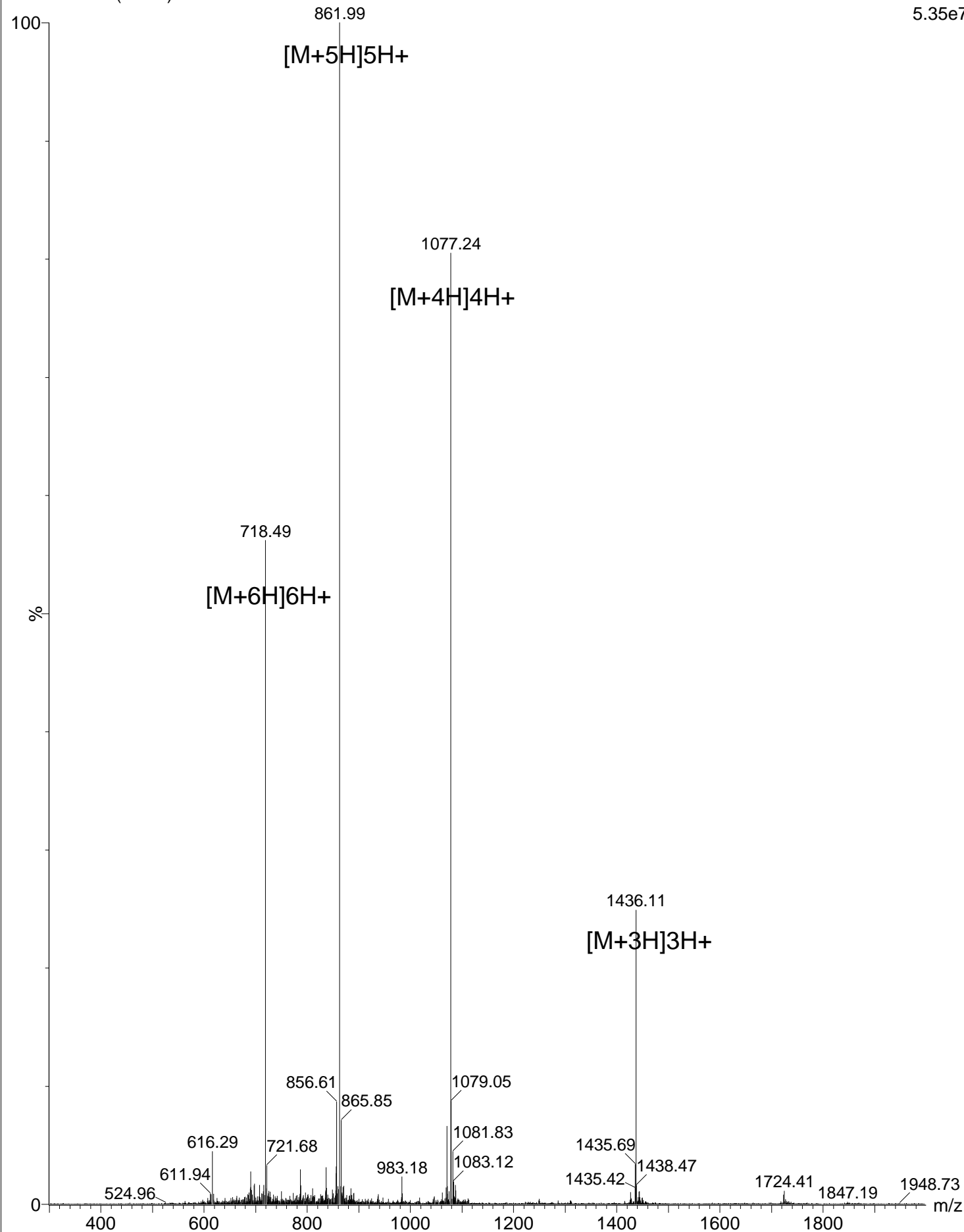

Supplement: Supplementary file 6 — Source Data [file 41467_2022_34009_MOESM6_ESM.zip › source data/biophysical analyses and purity assessment/LAPTH(I5H;A3S)-MS.pdf]

92740 P 3 (0.055)

Scan ES+  
3.68e6

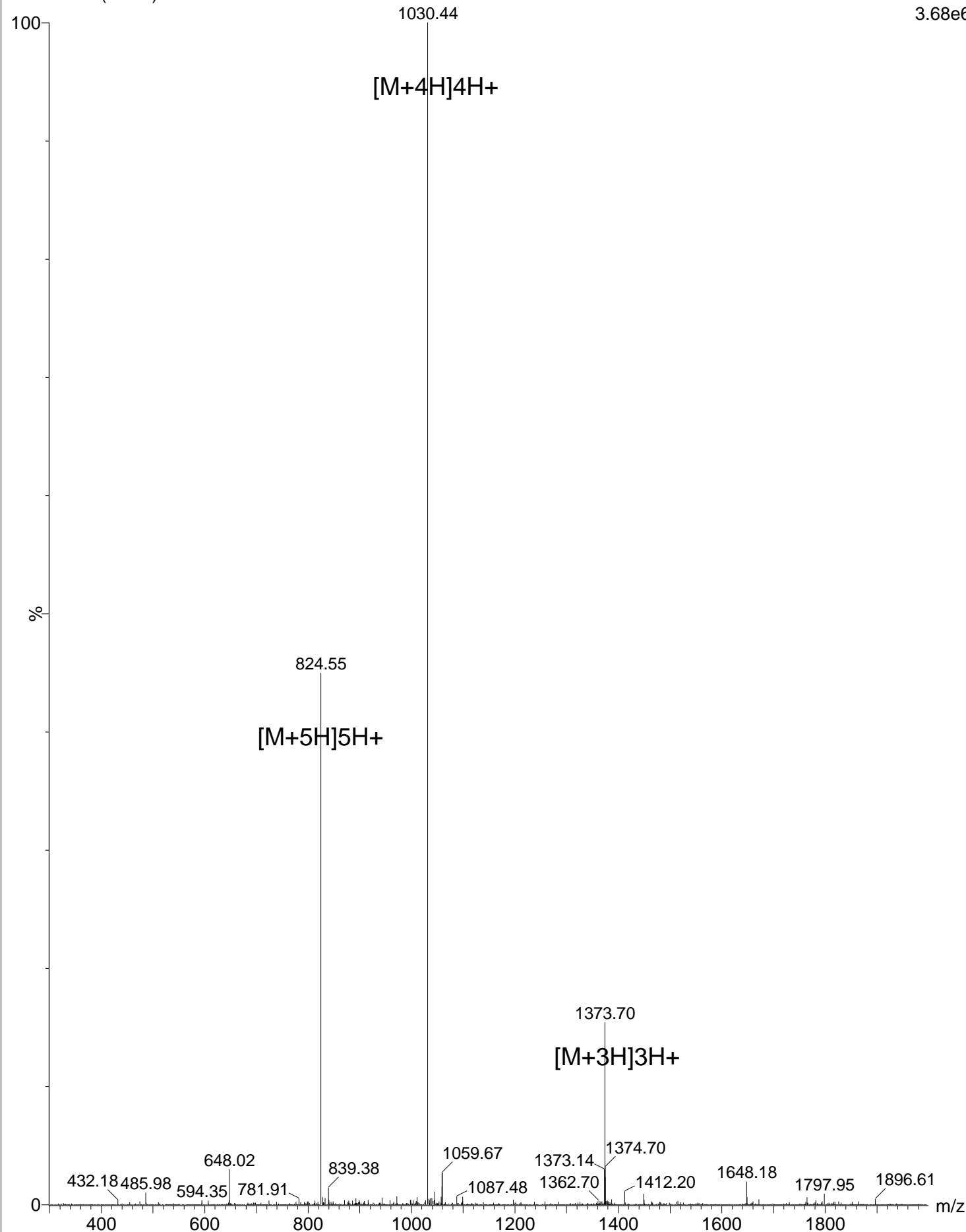

Supplement: Supplementary file 6 — Source Data [file 41467_2022_34009_MOESM6_ESM.zip › source data/biophysical analyses and purity assessment/PTH-MS.pdf]

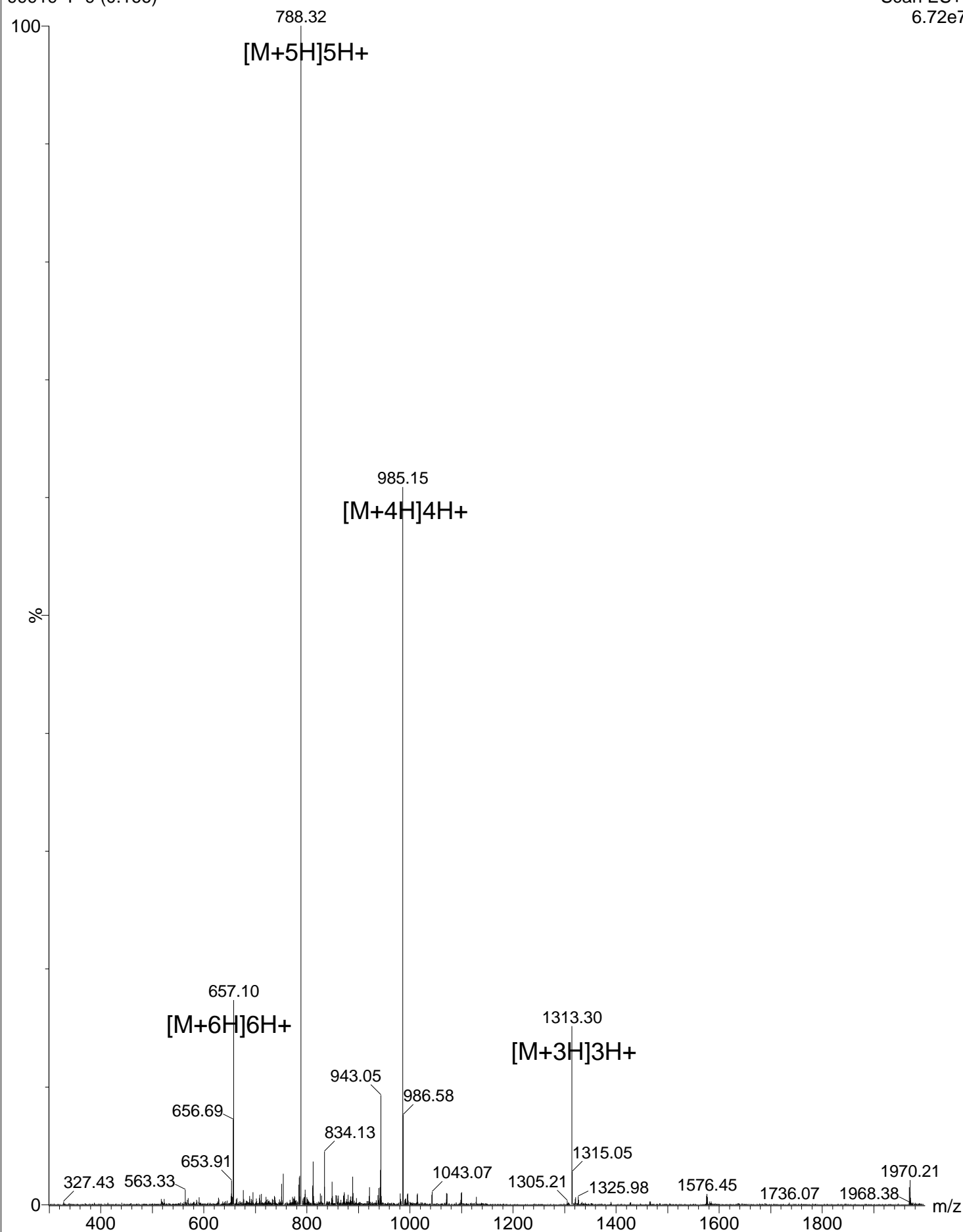

Supplement: Supplementary file 6 — Source Data [file 41467_2022_34009_MOESM6_ESM.zip › source data/biophysical analyses and purity assessment/ABL(H5I)-MS.pdf]

99819 P 6 (0.111)

Scan ES+  
5.83e7

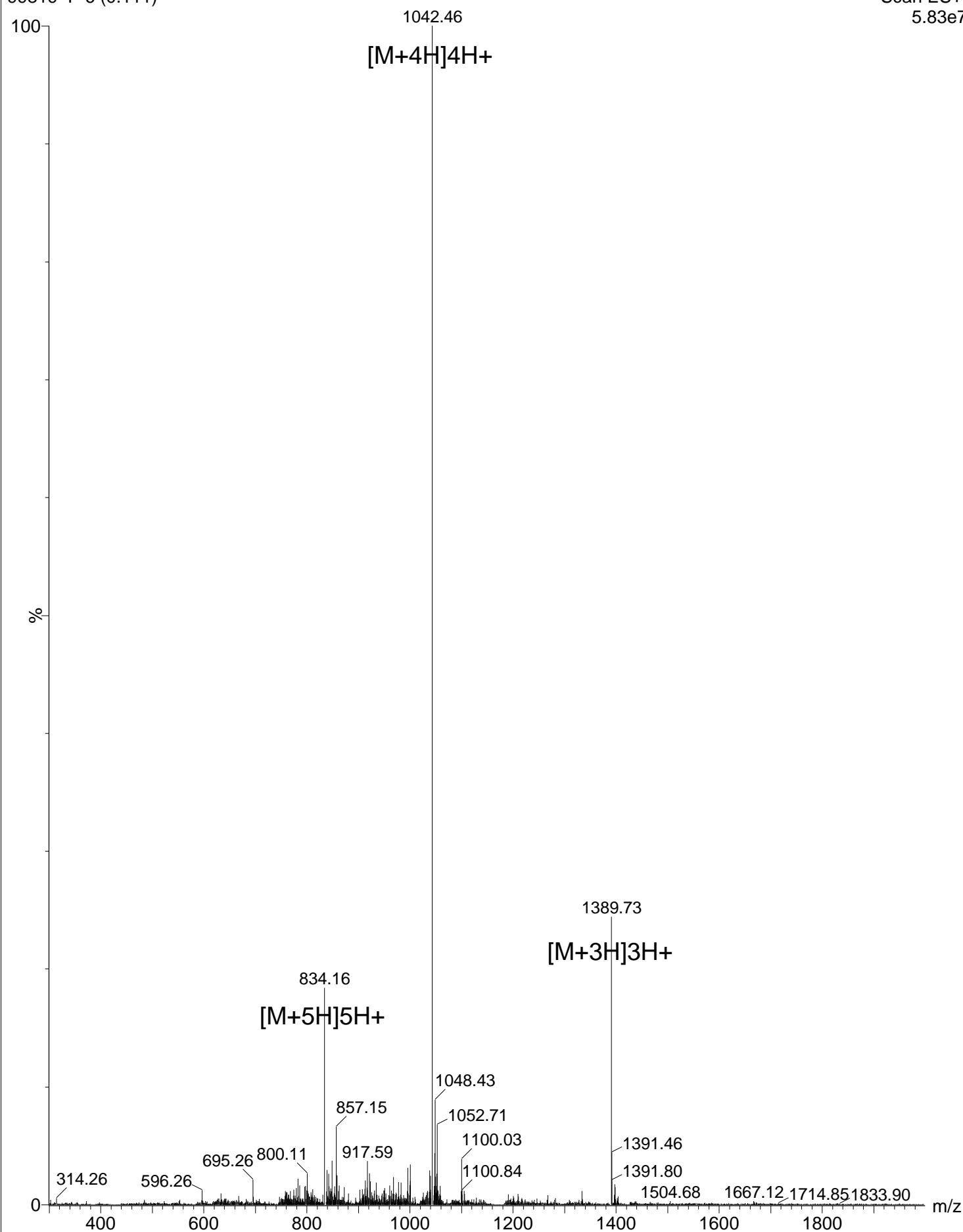

Supplement: Supplementary file 6 — Source Data [file 41467_2022_34009_MOESM6_ESM.zip › source data/biophysical analyses and purity assessment/LAPTH(W14S)-MS.pdf]

162632 P 7 (0.129)

Scan ES+  
1.36e7

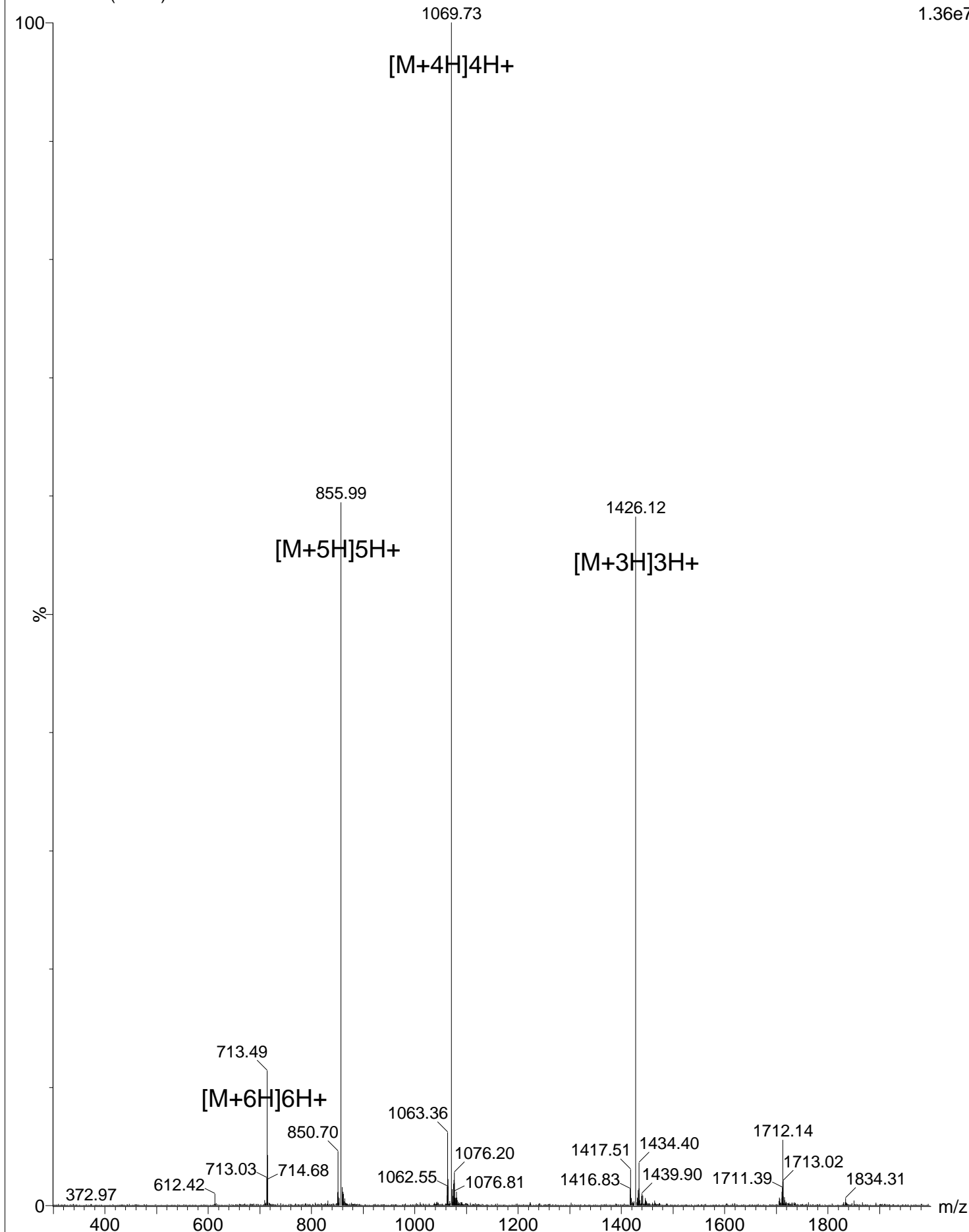

Supplement: Supplementary file 6 — Source Data [file 41467_2022_34009_MOESM6_ESM.zip › source data/biophysical analyses and purity assessment/LAPTH(I5H;A12G)-MS.pdf]
